# Slow Relaxation of Photogenerated Charge Carriers Boosts Open-Circuit Voltage of Organic Solar Cells

**DOI:** 10.1021/acs.jpclett.1c02235

**Published:** 2021-10-05

**Authors:** Tanvi Upreti, Sebastian Wilken, Huotian Zhang, Martijn Kemerink

**Affiliations:** † Complex Materials and Devices, Department of Physics, Chemistry and Biology (IFM), Linköping University, 581 83 Linköping, Sweden; ‡ Centre for Advanced Materials, Heidelberg University, Im Neuenheimer Feld 225, 69120 Heidelberg, Germany; § Physics, Faculty of Science and Engineering, Åbo Akademi University, Porthansgatan 3, 20500 Turku, Finland; ∥ Biomolecular and Organic Electronics, Department of Physics, Chemistry and Biology (IFM), Linköping University, 581 83 Linköping, Sweden

## Abstract

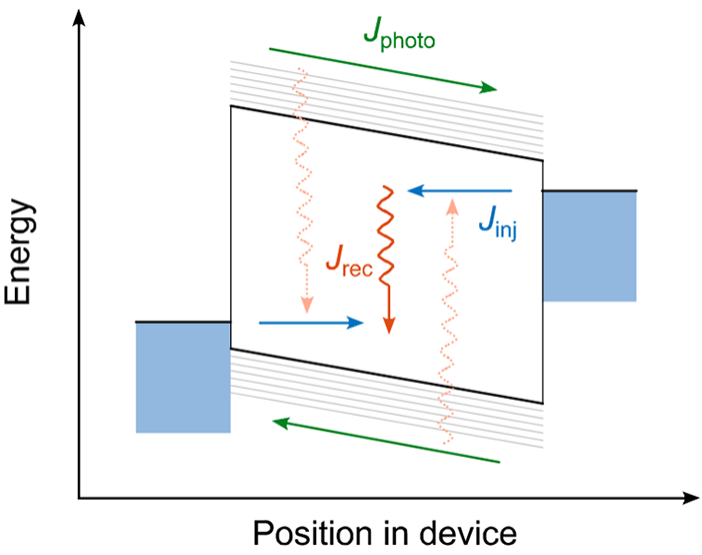

Among the parameters determining the efficiency of an organic solar cell, the open-circuit voltage (*V*_OC_) is the one with most room for improvement. Existing models for the description of *V*_OC_ assume that photogenerated charge carriers are thermalized. Here, we demonstrate that quasi-equilibrium concepts cannot fully describe *V*_OC_ of disordered organic devices. For two representative donor:acceptor blends, it is shown that *V*_OC_ is actually 0.1–0.2 V higher than it would be if the system was in thermodynamic equilibrium. Extensive numerical modeling reveals that the excess energy is mainly due to incomplete relaxation in the disorder-broadened density of states. These findings indicate that organic solar cells work as nonequilibrium devices, in which part of the photon excess energy is harvested in the form of an enhanced *V*_OC_.

Organic photovoltaics (OPVs) achieve quantum yields^1^ and fill factors (FFs)^2,3^ that are competitive with established technologies such as crystalline Si and GaAs. However, the situation is different with the open-circuit voltage (*V*_OC_). Relative to the energy of the photons absorbed, *V*_OC_ is low in OPVs, with the consequence that the overall efficiency lags behind their inorganic counterparts.^4^ This makes understanding the nature of these voltage losses and finding strategies to reduce them essential research problems regarding OPVs.

According to current understanding, *V*_OC_ is generally limited by the splitting of the quasi-Fermi levels of electrons and holes under illumination.^5^ Using the principle of detailed balance gives
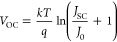
1where *k* is the Boltzmann constant, *T* the temperature, *q* the elementary charge, *J*_SC_ the short-circuit current, and *J*_0_ the dark saturation current. The parameter *J*_0_ reflects the current associated with thermal excitation of electrons over the band gap; it thus contains all information about recombination losses, via either radiative or nonradiative pathways.^6,7^ Using the reciprocity relation by Rau,^8^ we can calculate *J*_0_ from the photovoltaic quantum efficiency EQE_PV_ and the electroluminescence (EL) quantum efficiency EQE_EL_ via
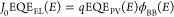
2Here, *E* is the photon energy and *ϕ*_BB_ the blackbody spectrum at a given temperature.^9^ It follows from eqs 1 and 2 that *V*_OC_ is maximized when *J*_0_ is in its thermodynamic limit, that is, when EQE_EL_ equals unity and all recombination is radiative.

As compared to the ideal situation in the Shockley–Queisser model, typically three loss channels are considered in OPVs. First, the energetic driving force due to an energy level offset between electron donor and acceptor; even though these losses have been drastically reduced by the transition from fullerene to nonfullerene acceptors (NFAs),^10,11^ they may still amount to 0.2–0.3 V depending on the local energy landscape at the donor/acceptor interface.^12^ Second, nonradiative recombination, reducing *V*_OC_ by a factor *kT*/*q* ln(EQE_EL_), which is estimated to be at least about 0.2 V even in the best OPVs to date.^13,14^ Third, energetic disorder in the density of states (DOS); it has been shown that the higher the disorder (given by the width σ of the DOS), the lower *V*_OC_, since carriers sink deeper into the DOS.^15^

The concepts outlined above have in common that they—either explicitly or implicitly—assume photogenerated carriers to be in thermal equilibrium with the lattice. This assumption is well justified for inorganic semiconductors, where band transport dominates and thermalization occurs by phonon emission on subpicosecond time scales, such that carriers are transported at quasi-equilibrium energies.^5^ In organic materials, relaxation is more complicated and consists of two distinct processes. First, a fast, subpicosecond thermalization by coupling to molecular vibrations brings the system to the lowest excited state.^16,17^ Because the local site energy is typically not the global energy minimum, a second thermalization occurs via thermally activated tunneling (hopping) in the typically broad distribution of localized sites, which is slow.^18^ Experimental and numerical studies have shown that excess carriers in OPVs are collected before this second process has completed, that is, before photogenerated charges are fully relaxed in their respective DOS.^19,20^ Also, the distribution of charge transfer (CT) states that form under steady-state illumination was shown to be characterized by an effective temperature that exceeds the temperature of the ambient.^21^ Recent work provides evidence that also the EL of the interfacial CT state is governed by nonequilibrium effects.^22^ However, surprisingly little is known about how slow thermalization in OPVs affects the device *V*_OC_.

Here, we show that the *V*_OC_ of disordered OPVs can significantly exceed its equilibrium value. We demonstrate this for two material systems: a traditional polymer:fullerene blend and a recent polymer:NFA blend with 16% efficiency. In both cases, the experimental *V*_OC_ is 0.1–0.2 V higher than predicted by quasi-equilibrium device simulations and by eq 1 with input parameters from the reciprocity analysis.^8,23^ Instead, using an experimentally calibrated kinetic Monte Carlo (KMC) model^24^ gives a good description of the device *V*_OC_ as well as its dependence on thickness and temperature. With this, we show that the excess energy due to incomplete thermalization can actually be harvested, and we propose that disordered OPVs can work as “hot” carrier solar cells.

We will first focus on TQ1:PC_71_BM blends, for which the importance of nonequilibrium effects is well documented.^19,20,22^ Recently, we have developed a KMC model that can describe current–voltage (*J*–*V*) curves of complete OPVs and predict, as opposed to fit, the device *V*_OC_, *J*_SC_, and FF under illumination as a function of, for example, thickness and temperature.^24^Figure 1 demonstrates this for a TQ1:PC_71_BM solar cell with an active-layer thickness of 70 nm. The model fully accounts for slow relaxation in the disorder-broadened DOS (assumed to be Gaussian in shape) and makes experimentally justified assumptions about the carrier injection at the contacts and the CT recombination rate. Furthermore, it implements a minimalistic but sufficiently realistic model of the morphology, consisting of a molecularly mixed TQ1:PC_71_BM matrix with embedded PC_71_BM aggregates.^24^ The model and the used parameters are further discussed in the Supporting Information (section 2).

**Figure 1:**
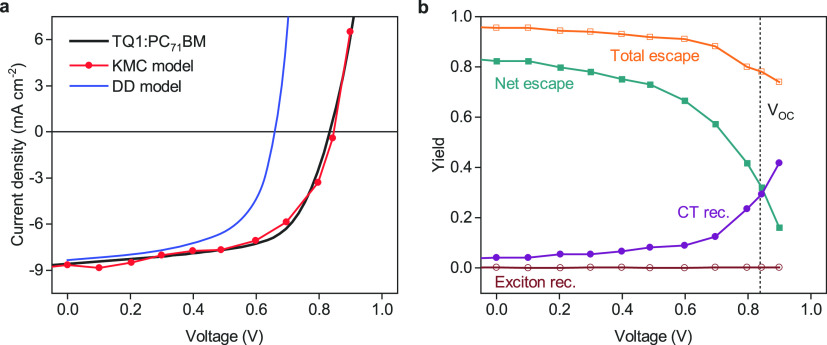
Measured versus modeled *J*–*V* curves and loss analysis. (a) The black line represents measured *J*–*V* characteristics of a 70 nm thick TQ1:PC_71_BM solar cell under illumination. Only the KMC model (red symbols) gives an accurate description of the experiment, while the DD model (blue line) that inherently assumes thermal equilibrium underestimates *V*_OC_ by about 0.2 V. Both models use a single, consistent set of parameters. (b) Corresponding extraction and loss yields from KMC. Total and net escape yields are defined as *y*_total_ = (*J*_*n*,an_ + *J*_*n*,cat_ + *J*_*p*,an_ + *J*_p,cat_)/*J*_abs_ and *y*_net_ = (−*J*_*n*,an_ + *J*_*n*,cat_ + *J*_*p*,an_ – *J*_*p*,cat_)/*J*_abs_, where *J*_(*n/p*),(an*/*cat)_ is the current density of photogenerated electrons/holes extracted via the anode/cathode and *J*_abs_ is the current density corresponding to light absorption. The curves labeled exciton and CT recombination show the relative current densities associated with exciton and CT recombination, that is, the fraction of photogenerated charges that undergo these processes. Similar data for the DD model can be found in the Supporting Information (section 9).

It is, in this context, important that KMC is the gold standard for charge transport simulations in these types of materials. Although drift–diffusion (DD) models can reproduce KMC results in certain cases (e.g., space charge limited transport^25^) when the right mobility functionals and boundary conditions are used, DD is a simplification that, among other aspects, upfront assumes charge carrier populations to be fully thermalized. Hence, to find out how the simplifications in DD work out in the case of OPVs, we calibrated the KMC model to the experiments and used the obtained parameters as input for a DD model. In particular, in the DD model we assumed the same injection barriers and the same energy gap between the highest occupied molecular orbital (HOMO) of the donor and the lowest unoccupied molecular orbital (LUMO) of the acceptor as in the KMC model, while energetic disorder was implemented via established mobility functionals.^25^ In other words, both models are used to describe the very same device, with the crucial difference that in the KMC model, nonequilibrium effects are accounted for, whereas the DD formalism is inherently based on the assumption of near-equilibrium through the use of Boltzmann statistics. In the limit that nonequilibrium effects are unimportant, DD simulations with parametrized mobilities, as used here, accurately reproduce the more detailed KMC calculations.^25^ Full details of the simulations are provided in the Supporting Information. As can be seen from the blue line in Figure 1, the DD model describes *J*_SC_ and the shape of the *J*–*V* curve reasonably well, but significantly underestimates *V*_OC_ by about 0.2 V. This clearly shows that the equilibrium DD approach does not capture all relevant physics.

To test the general validity of our statement and to highlight the predictive value of our KMC model, we fabricated OPVs with varying active-layer thickness. Thickness variations allow probing the device characteristics for a range of extraction times and carrier densities, and intuitively, one might expect nontrivial behavior for systems where extraction competes with thermalization. The variation of the generation rate profile with thickness was explicitly taken into account via transfer-matrix modeling.^26^Figure 2 shows that while the KMC model provides an excellent description of the device *V*_OC_, the mismatch between experiment and DD model remains independent of the thickness. The trend in *V*_OC_ predicted by the DD model is in good agreement with the analytical model by Blakesley and Neher,^15^ which supposes full relaxation to effectively reduce the HOMO–LUMO gap by σ^2^/*kT*. Specifically, in the DD model, *V*_OC_ follows the modulation in charge generation rate (and thus *J*_SC_) due to interference in the multilayer device, as suggested by eq 1. This strict correlation is observed neither in the experiment nor in the KMC simulation, which suggests that *V*_OC_ results from a more complex interplay between generation (profiles) and thermalization. The absence of a clear decreasing trend in *V*_OC_ with increasing thickness might appear at odds with the notion of an ongoing and incomplete thermalization. However, thermalization in disordered media follows roughly a log–linear time dependence until an equilibrium energy is reached.^18^ The implication of that is that the differences in extraction time for experimentally achievable thickness variations only cause minor differences in thermalization and are overwhelmed by other effects like changes in absorption and recombination.

**Figure 2:**
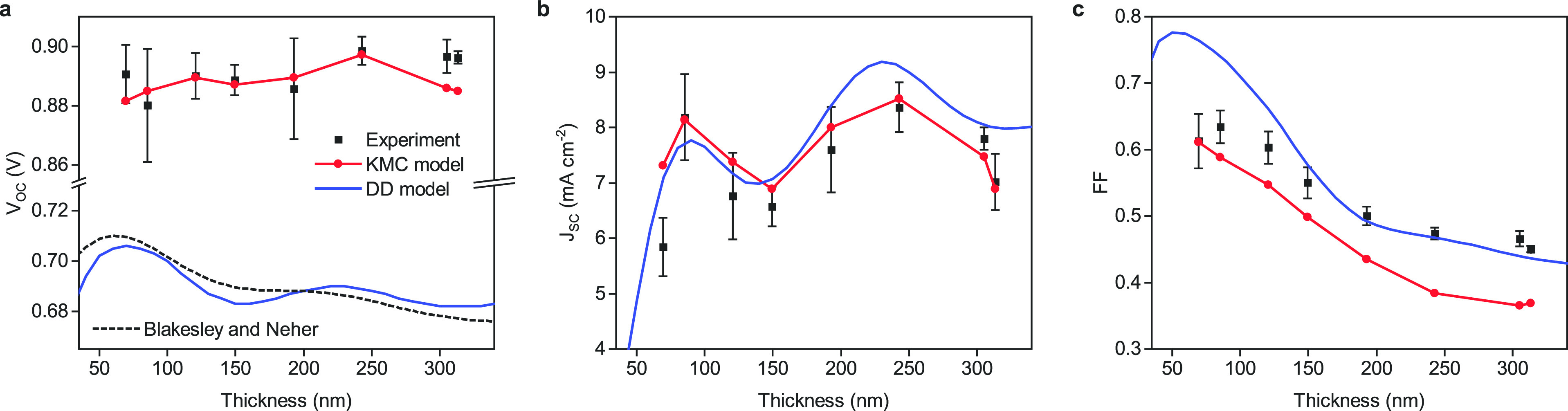
Thickness-dependent device performance. Shown is the (a) open-circuit voltage, (b) short-circuit current, and (c) fill factor of TQ1:PC_71_BM solar cells with varying active-layer thickness (black symbols) together with the predictions of the KMC model (red symbols) and the DD model (blue lines). The dashed line in (a) represents the analytical model by Blakesley and Neher^15^ assuming carriers to be fully relaxed.

In contrast to *V*_OC_, both models show a good agreement with the experimental *J*_SC_ and, to a lesser degree, the FF (see Figures 2b and 2c, respectively). Hence, the kinetic competition between extraction and recombination is also reasonably described by the DD model.^27,28^ This is especially the case for the thick devices, where the FF approaches its space-charge limit due to imbalanced transport.^29,30^ We note that the limitations of DD modeling regarding *V*_OC_ can be partly overcome without losing accuracy in *J*_SC_ and FF by artificially increasing the band gap, but this would be an ad hoc compensation of the inability of the DD model to capture nonequilibrium effects and lead to an inconsistency between the parameters in the two models. The fact that the band gap effectively acts as a fit parameter provides a plausible explanation why DD modeling is so successful in describing *J*–*V* curves of OPVs. It also confirms that it can indeed lead to useful and valid results when looking at certain macroscopic phenomena such as space charge or carrier injection and extraction. However, the point we want to make here is that when dealing with questions about the nature and limiting factors of *V*_OC_, DD models provide an incomplete picture of the physical reality in OPVs.

We recently demonstrated that our KMC model can also be used to accurately describe the spectral shape and position of the solar cell’s absorption and emission.^22,31^ This gives us the opportunity to estimate *V*_OC_ from eq 1 with the KMC input parameters used in Figure 1, assuming strict equilibrium conditions. The procedure is detailed in the Supporting Information (section 3) and gives *V*_OC_ ≈ 0.69 V. Despite the simplifications made, this value is strikingly close to the value of *V*_OC_ ≈ 0.66 V found in the DD simulations in which Boltzmann statistics, that is, near-equilibrium conditions, are implicitly assumed. Hence, the DD and the analytical model consistently show that *V*_OC_ would be ∼0.2 V lower than actually measured if the device was operating in thermal equilibrium. In other words, voltage losses in this OPV system would be significantly larger if thermalization would complete in the charge carrier lifetime. The device operates as a hot carrier solar cell.^32−34^ In the Supporting Information (section 4), we confirm the result from our earlier work^19^ that the charge carrier populations do not reach equilibrium prior to extraction.

Another indication that far-from-equilibrium charges contribute significantly to the *J*–*V* curve under illumination comes from the incomplete saturation of the photocurrent at short circuit (*V* = 0). This is just visible in Figure 1a and more clearly in Figure S7 of the Supporting Information, where we also show that the KMC model does and the DD model does not reproduce the observation. The cause for this difference becomes clear from the loss analysis in Figure 1b and the corresponding data in Figure. S7. First, the nearly complete saturation of the CT recombination yield already at small forward bias (cf. Figure 1b) rules out field-dependent charge generation as an explanation.^35^ Instead, the large difference between net and total escape yields at short-circuit conditions indicates that despite the ∼1 V built-in field, a large fraction of photogenerated charges leave the device via the wrong (nonselective) contact at short circuit. This points to highly diffusive, nonequilibrium charge motion, requiring large fields to be suppressed. The strong voltage dependency of the difference between net and total escape yields confirms this notion. Such a diffusion-loss scenario is fully in line with our earlier analysis of ultrafast transport and absorption.^19,20,22^

Another important conclusion that can be drawn from the total and net escape yields in Figure 1b is that at *V*_OC_, about 80% of the photogenerated charges do not recombine but instead are extracted from one of the contacts (total escape yield). Because ∼30% of the photogenerated charges are extracted at the desired contact (net escape yield), there is a net photocurrent. Because *J* = 0 at *V*_OC_, there must be a balancing injection current corresponding to ∼30% of the short-circuit current. The existence of such an injection current is hard, if not impossible, to measure directly. However, a strong indication for this happening comes from the recent observation that recombination kinetics around *V*_OC_ in this system follow near-equilibrium rates:^36^ because under *V*_OC_ conditions the vast majority of “nonequilibrium” photogenerated charges still leaves the device via the contacts, recombination is likely to be governed by “equilibrium” charges being injected from the contacts that act as thermal reservoirs. On top of this comes the small fraction of “thermalized” photogenerated charges that does get trapped in the disordered DOS. The above considerations are condensed in the cartoon shown in Figure 3 and are further discussed in the Supporting Information (section 4) where it is shown that even at open-circuit conditions, a high-energy (nonthermalized) photocurrent runs through the device, as argued above and indicated in Figure 3.

**Figure 3:**
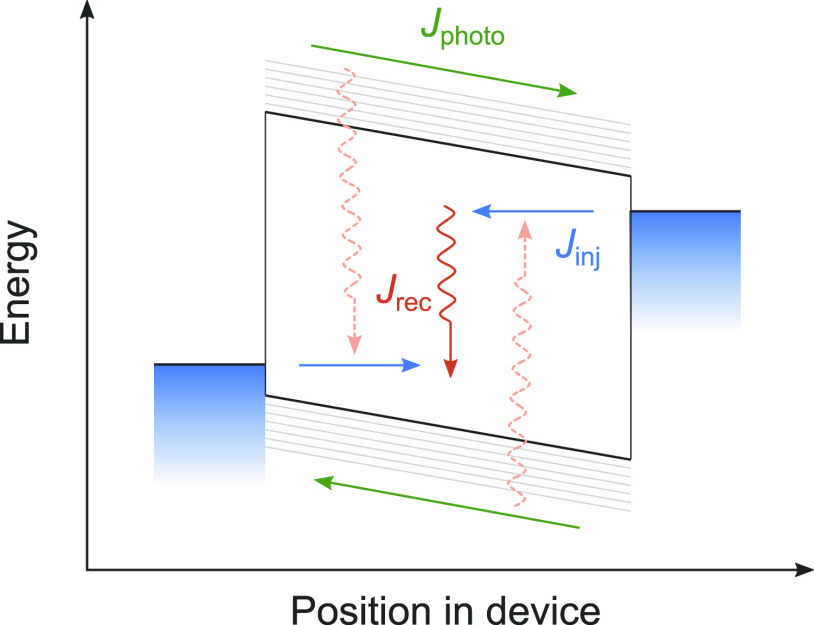
Schematic charge carrier kinetics in an operational OPV device at open-circuit conditions. Nonthermalized photocurrents (*J*_photo_, green arrows) and thermalized injection currents (*J*_inj_, blue arrows) balance. Recombination (corresponding current: *J*_rec_) is weak for photogenerated charges, but strong for injected charges. The gradual but incomplete thermalization of the photocharges is not shown.

The excess energy of photogenerated charges due to the incomplete thermalization during extraction forms a reservoir of energy. Equivalently, it can be considered as a (time-dependent) effective temperature of the charge carrier distribution that significantly exceeds the lattice temperature.^37^ This introduces another relevant energy scale in the system, and one may anticipate a pronounced effect on the temperature dependence of the device characteristics. Figure 4a,b shows the temperature-dependent *J*–*V* behavior for a relatively thin TQ1:PC_71_BM device. Similar data for a thicker device is shown in Figure S10. Clearly, the KMC model (using the same parameters as before) provides a reasonable description of the experiment at all temperatures, confirming that it captures all essential device physics. This is in stark contrast to the DD model, which not only underestimates *V*_OC_ regardless of temperature, but also fails to capture the temperature dependence of the shape of the *J*–*V* curves, as shown in Figure S8. Nevertheless, the slope of *V*_OC_ versus temperature is very comparable in both KMC and DD simulations, and the same holds for the forward part of the *J*–*V* curves. We attribute this to the fact that in both models the temperature dependence of *V*_OC_ is dominated by the strong temperature dependence of the forward injection current that, as discussed in the context of Figure 3, is carried by a charge carrier population that reflects the equilibrium temperature.

**Figure 4:**
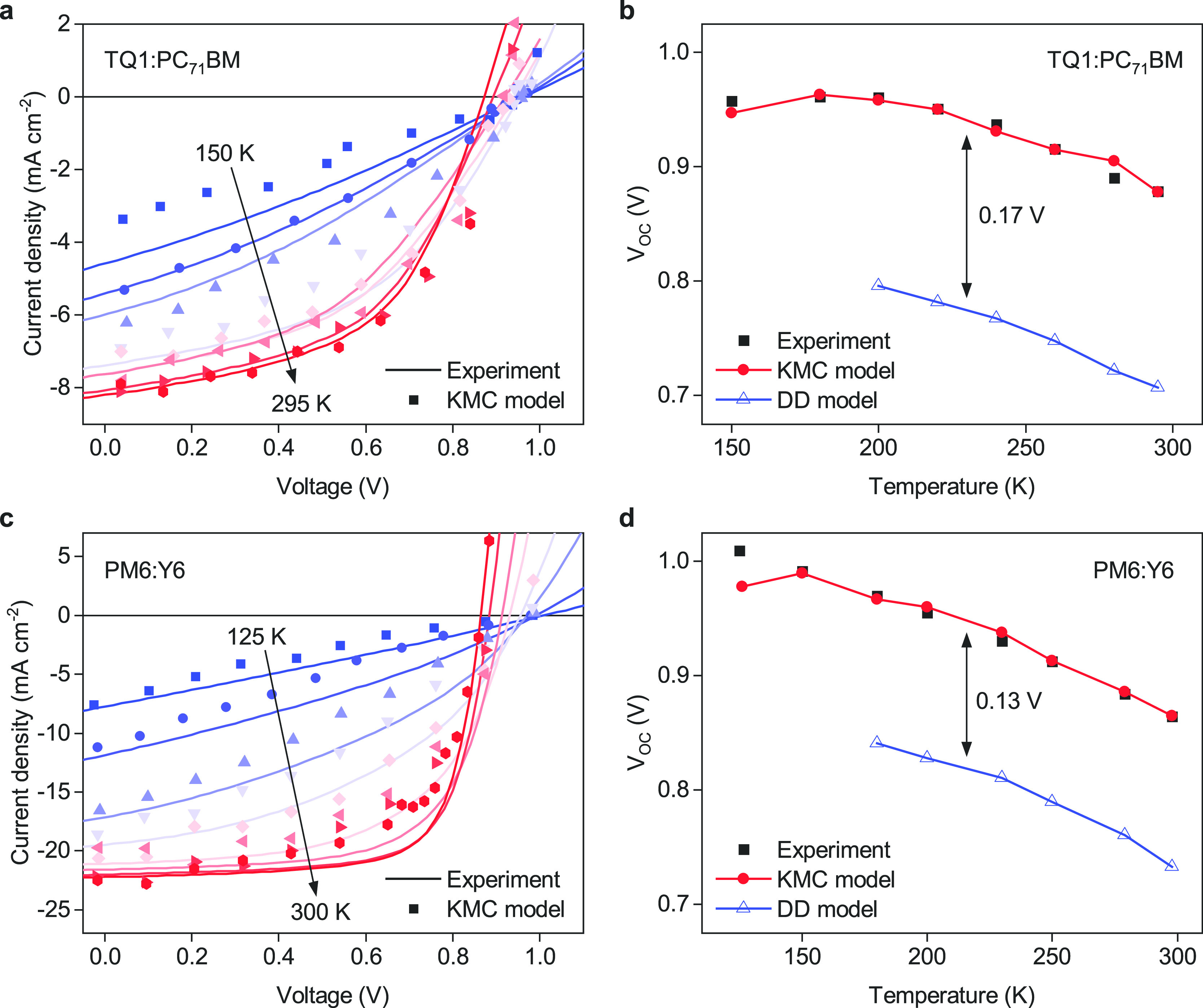
Temperature-dependent device performance. Panels a and c show temperature-dependent *J*–*V* curves for a 75 nm thick TQ1:PC_71_BM solar cell and a 115 nm thick PM6:Y6 solar cell, respectively. Solid lines are experimental data and symbols are KMC simulations. Panels b and d show the corresponding values of *V*_OC_ and compare them with the results of the DD model.

So far, we have shown that a relatively amorphous and disordered polymer:fullerene OPV system should be treated as a far-from-equilibrium hot carrier device. An important question is whether or not the same holds true for modern NFA systems. To this end, we performed a similar series of experiments on the state-of-the-art PM6:Y6 system that provides power conversion efficiencies over 15%.^10,38^ In a recent work, we investigated the energetic disorder in a range of OPV blends and found Gaussian disorder values σ ranging from 45 to 80 meV, without any clear correlation between σ and *V*_OC_.^39^ For the PM6:Y6 system, σ_HOMO_ ≈ 89 meV and σ_LUMO_ ≈ 68 meV were found. Because these numbers are much higher than the thermal energy *kT* ≈ 25 meV at room temperature and well exceed the thresholds we previously found for disorder to become negligible,^20^ one should expect the phenomena discussed above to be relevant for this NFA system as well. Figure 4c,d shows the temperature dependence for a 115 nm thick PM6:Y6 solar cell. Although the difference with the KMC model is smaller than for the more disordered TQ1:PC_71_BM system, also the highly efficient PM6:Y6 system cannot be correctly described by the DD model (see also Figure S9), while the KMC model reproduces the experiment reasonably well, especially regarding the temperature dependence of *V*_OC_. Again, consistent input parameters were used for DD and KMC modeling (Supporting Information, section 2). This shows that also for NFA systems with relatively low disorder and balanced electron and hole mobilities, *V*_OC_ is insufficiently described by equilibrium concepts. A further discussion of the role of disorder on the *V*_OC_ difference between KMC and DD is given in the Supporting Information (section 8).

As shown in Figure 5, a remarkable difference between KMC and DD simulations is in the dark currents that show an upswing that is shifted by essentially the same amount as *V*_OC_, despite the fact that the very same boundary conditions are used (Supporting Information, section 2). This difference cannot be attributed to nonequilibrium effects since in the dark all charges are injected from thermalized reservoirs instead of being photogenerated. The dark *J*–*V* curves are consistent with those under illumination in the sense that the superposition principle *J*_light_ ≈ *J*_dark_ – *J*_SC_ is obeyed. Approximating the dark current with the Shockley equation, *J*_dark_ = *J*_0_(exp(*qV*/*kT*) – 1), this means that the dark reverse saturation current *J*_0_ must be different in both models. Above we discussed how for DD *J*_0_ and concomitantly *V*_OC_ can be calculated from the model input parameters as the overlap of the EQE_PV_ spectrum with the blackbody spectrum (see also Supporting Information, section 3). To save a near-equilibrium interpretation of *V*_OC_ in the case of KMC, one would have to find a meaningful alternative way of calculating *J*_0_. In the used framework, the most logical and in fact only viable way is to assume that part of the absorption spectrum does not contribute to the EQE, which implies an IQE that drops to zero below some threshold energy. For the TQ1:PC_71_BM system, such behavior has indeed been observed.^31^ However, to reproduce the *V*_OC_ = 0.88 V value from KMC, one would have to cut the absorption spectrum below ∼1.12 eV. Doing so produces the dashed gray line in Figure 5a. However, following the method laid out in our earlier work,^31^ we calculated the energy-dependent IQE for the parameters used (see Figure 5b). Clearly, the true IQE spectrum does not roll off at 1.12 eV but around 0.86 eV. Using the empirical function shown by the red line as IQE(*E*) leads to a *V*_OC_ around 0.73 V, as shown by the solid gray line in Figure 5a, which is somewhat higher than found for unity IQE, but still far of the actual *V*_OC_. The observed inconsistency between the fitted IQE spectrum and the actual IQE spectrum (gray area in Figure 5b) points toward a fundamental problem in the used reciprocity formalism when applied to organic solar cells. Specifically, the assumed equivalence of injection and extraction will be violated when one of the channels is far from equilibrium while the other is not.^23^

**Figure 5:**
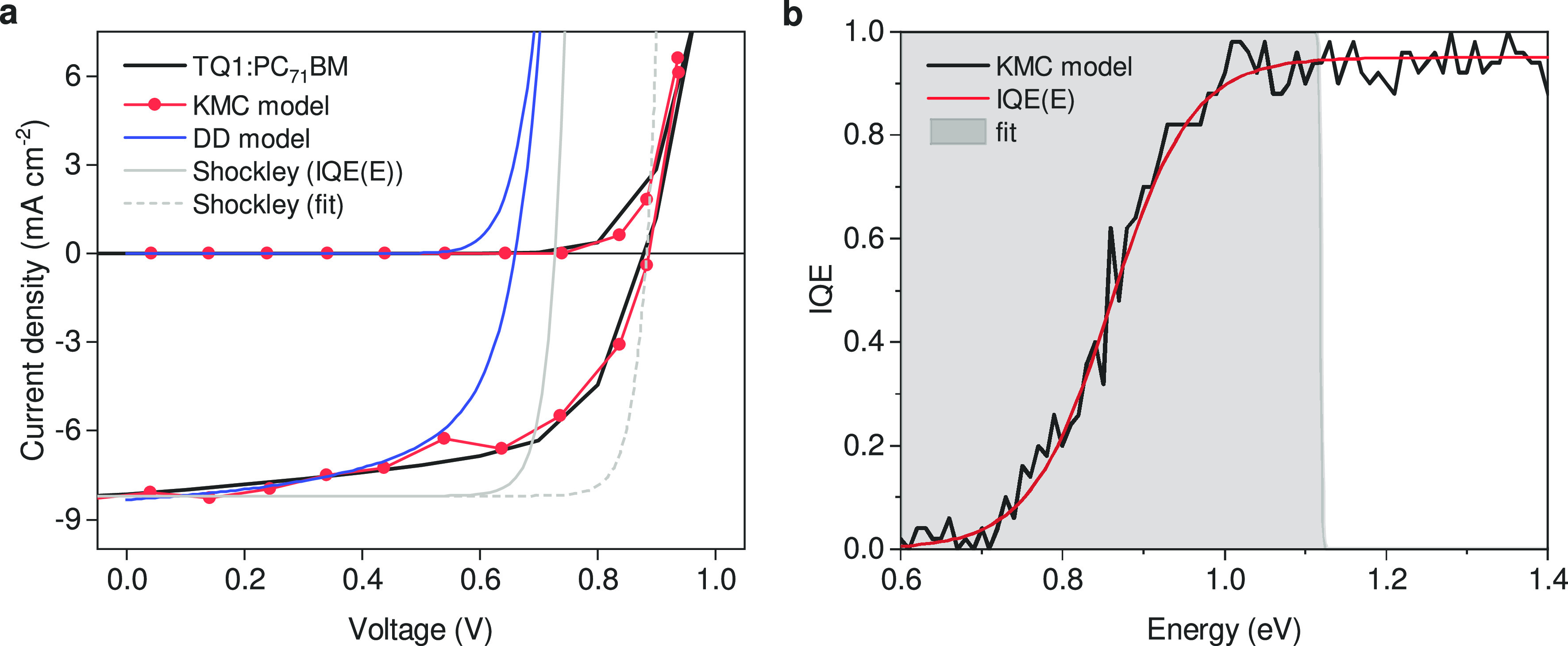
Analysis of difference between DD and KMC models. (a) *J*–*V* curves in dark and light (solid black lines) compared to DD (blue lines) and KMC (red symbols). The shown sample is nominally identical with that in Figure 1; the simulations are the same, but slightly rescaled to match *J*_SC_. The gray lines are calculated from the Shockley equation, *J* = *J*_0_(exp(*qV*/*kT*) – 1), with different *J*_0_ as explained in the text. (b) IQE calculated by KMC (black line) and an empirical IQE(*E*) function (red line). The gray shaded area is explained in the text.

The above strong indications for the importance of nonequilibrium kinetics for the performance of two prototypical OPV systems naturally raise the question why previous analysis in terms of reciprocity relations, which are derived on the assumption that detailed balance holds, worked so well.^8,9,13,36,40,41^ Here, one has to make a distinction between at least two different uses of reciprocity relations. First, in many works reciprocity between emission and action spectra is used to convert one into the other with the aim to establish an estimate for the “relaxed” CT energy, which subsequently is used as a reference point for further analysis.^9,13,40,41^ In our previous work, we have shown that this reciprocity is not strictly obeyed due to the CT emission coming from a nonthermal subset of the full CT manifold.^22,31^ However, the typical energy range over which experiments can be analyzed makes it hard to pinpoint systematic deviations from the phenomenological reciprocity.

More interesting in the current context is the use of eqs 1 and 2 to predict *V*_OC_. It was shown by Roland et al.,^36^ for example, that experimentally measured EQE_EL_ and EQE_PV_ spectra can be used to accurately predict the open-circuit voltage of 150–200 nm thick TQ1:PC_71_BM devices. Although this topic warrants further investigation, we speculate that at least part of the answer is due to a cancellation of errors. In ref (31), *V*_OC_ is calculated as *V*_OC_ = *V*_OC,rad_ + (*kT*/*q*) ln(EQE_EL_) by using an EQE_EL_ in the range of 10^–5^–10^–6^, which is a common value for OPV systems and corresponds to a voltage loss around 0.3–0.35 V.^41^ As also argued in the Supporting Information (section 3), the reasonable assumption that CT and S_1_ recombination are competing against the same or at least similar loss channels yields that their relative lifetimes, and therefore their relative EQE_EL_, should reflect their relative oscillator strengths. Interestingly, for the few OPV systems for which CT lifetime estimates are known, it differs by 1–2 orders of magnitude from the S_1_ lifetime that is typically in the nanoseconds range in OPVs.^42^ This ratio is consistent with the typical difference in CT and S_1_ absorption strengths,^31,36,40^ but not with an EQE_EL_ for CT recombination of 10^–5^–10^–6^. Using instead an EQE_EL_ around 10^–2^, that is, the approximate ratio of the S_1_ and CT lifetime, leads to a voltage loss of ∼0.12 V, which happens to differ from the original 0.3–0.35 V voltage loss by an amount that is rather similar to the ∼0.2 V difference in *V*_OC_ that we found between our KMC simulations and the reciprocity prediction in the Supporting Information (section 3). A concise overview of the differences between equilibrium and nonequilibrium interpretations of *V*_OC_ is given in the same section of the Supporting Information.

A possible partial explanation for the severe underestimation of the EQE_EL_ in experiments is that it is tacitly assumed that all injected charges at voltages corresponding to *V*_OC_ recombine. This need not be the case in carefully optimized OPV morphologies that consist of phase-separated donor and acceptor domains. In such morphologies, which are designed to efficiently keep electrons and holes apart, injected electron and hole currents are likely to never meet. This is especially the case in blends where mixed and pure regions coexist, resulting in an energy cascade that pushes charge carriers away from the donor/acceptor interface.^43^ In our KMC simulations for the TQ1:PC_71_BM system, this recombination fraction is ∼10^–1^, which is an upper limit since the simplified morphology used leads to a significant underestimation of the fill factor for thicker devices (Figure 2c).

Summarizing, we have shown for two exemplary OPV systems, a polymer:fullerene and polymer:NFA blend, that *V*_OC_ significantly exceeds its equilibrium value by 0.1–0.2 V. The excess energy arises because charge carriers are not completely relaxed in their disorder-broadened DOS when they are extracted at the contacts. Our results indicate that even under *V*_OC_ conditions, most of the photogenerated charge carriers do not recombine, but leave the device via one of the contacts. Instead, recombination is largely dominated by thermalized injected carriers, which explains the success of equilibrium concepts in the past. It should be noted that the higher *V*_OC_ does not necessarily translate into higher efficiency, as the latter depends on all three of the parameters *V*_OC_, *J*_SC_, and FF. Whether the nonequilibrium effects can be exploited to break the Shockley–Queisser limit or to realize OPVs with significantly higher film thicknesses is an interesting direction for future research.^44^ Because the material systems investigated here are not exceptional in terms of energetic disorder, but typical representatives of the state of the art, we expect that our results are highly relevant for most OPV systems.

## Supporting Information Available

All experimental and numerical details; additional experimental and numerical data; detailed discussions on the reciprocity analysis of KMC simulations, transient energetics of photogenerated charges, and the role of disorder (PDF)

## References

[ref1] Park S. H.; Roy A.; Beaupré S.; Cho S.; Coates N.; Moon J. S.; Moses D.; Leclerc M.; Lee K.; Heeger A. J. Bulk Heterojunction Solar Cells with Internal Quantum Efficiency Approaching 100%. Nat. Photonics 2009, 3 (5), 297–302. 10.1038/nphoton.2009.69.

[ref2] Gao W.; An Q.; Hao M.; Sun R.; Yuan J.; Zhang F.; Ma W.; Min J.; Yang C. Thick-Film Organic Solar Cells Achieving over 11% Efficiency and Nearly 70% Fill Factor at Thickness over 400 nm. Adv. Funct. Mater. 2020, 30 (10), 1908336 10.1002/adfm.201908336.

[ref3] Li Z.; Ying L.; Zhu P.; Zhong W.; Li N.; Liu F.; Huang F.; Cao Y. A Generic Green Solvent Concept Boosting the Power Conversion Efficiency of All-Polymer Solar Cells to 11%. Energy Environ. Sci. 2019, 12 (1), 157–163. 10.1039/C8EE02863J.

[ref4] Polman A.; Knight M.; Garnett E. C.; Ehrler B.; Sinke W. C. Photovoltaic Materials: Present Efficiencies and Future Challenges. Science 2016, 352 (6283), aad4424 10.1126/science.aad4424. 27081076

[ref5] Würfel P. Physics of Solar Cells: From Principles to New Concepts, 1st ed.; Wiley: 2005.

[ref6] Tvingstedt K.; Deibel C. Temperature Dependence of Ideality Factors in Organic Solar Cells and the Relation to Radiative Efficiency. Adv. Energy Mater. 2016, 6 (9), 1502230 10.1002/aenm.201502230.

[ref7] Cuevas A. The Recombination Parameter J0. Energy Procedia 2014, 55, 53–62. 10.1016/j.egypro.2014.08.073.

[ref8] Rau U. Reciprocity Relation between Photovoltaic Quantum Efficiency and Electroluminescent Emission of Solar Cells. Phys. Rev. B 2007, 76 (8), 085303 10.1103/PhysRevB.76.085303.

[ref9] Vandewal K.; Tvingstedt K.; Gadisa A.; Inganäs O.; Manca J. V. On the Origin of the Open-Circuit Voltage of Polymer–Fullerene Solar Cells. Nat. Mater. 2009, 8 (11), 904–909. 10.1038/nmat2548. 19820700

[ref10] Perdigón-Toro L.; Zhang H.; Markina A.; Yuan J.; Hosseini S. M.; Wolff C. M.; Zuo G.; Stolterfoht M.; Zou Y.; Gao F.; Andrienko D.; Shoaee S.; Neher D. Barrierless Free Charge Generation in the High-Performance PM6:Y6 Bulk Heterojunction Non-Fullerene Solar Cell. Adv. Mater. 2020, 32 (9), 1906763 10.1002/adma.201906763. 31975446

[ref11] Liu J.; Chen S.; Qian D.; Gautam B.; Yang G.; Zhao J.; Bergqvist J.; Zhang F.; Ma W.; Ade H.; Inganäs O.; Gundogdu K.; Gao F.; Yan H. Fast Charge Separation in a Non-Fullerene Organic Solar Cell with a Small Driving Force. Nat. Energy 2016, 1 (7), 16089 10.1038/nenergy.2016.89.

[ref12] Nakano K.; Chen Y.; Xiao B.; Han W.; Huang J.; Yoshida H.; Zhou E.; Tajima K. Anatomy of the Energetic Driving Force for Charge Generation in Organic Solar Cells. Nat. Commun. 2019, 10 (1), 2520 10.1038/s41467-019-10434-3. 31175294 PMC6555791

[ref13] Benduhn J.; Tvingstedt K.; Piersimoni F.; Ullbrich S.; Fan Y.; Tropiano M.; McGarry K. A.; Zeika O.; Riede M. K.; Douglas C. J.; Barlow S.; Marder S. R.; Neher D.; Spoltore D.; Vandewal K. Intrinsic Non-Radiative Voltage Losses in Fullerene-Based Organic Solar Cells. Nat. Energy 2017, 2, 17053 10.1038/nenergy.2017.53.

[ref14] Liu S.; Yuan J.; Deng W.; Luo M.; Xie Y.; Liang Q.; Zou Y.; He Z.; Wu H.; Cao Y. High-Efficiency Organic Solar Cells with Low Non-Radiative Recombination Loss and Low Energetic Disorder. Nat. Photonics 2020, 14 (5), 300–305. 10.1038/s41566-019-0573-5.

[ref15] Blakesley J. C.; Neher D. Relationship between Energetic Disorder and Open-Circuit Voltage in Bulk Heterojunction Organic Solar Cells. Phys. Rev. B 2011, 84 (7), 075210 10.1103/PhysRevB.84.075210.

[ref16] Němec H.; Nienhuys H.-K.; Perzon E.; Zhang F.; Inganäs O.; Kužel P.; Sundström V. Ultrafast Conductivity in a Low-Band-Gap Polyphenylene and Fullerene Blend Studied by Terahertz Spectroscopy. Phys. Rev. B 2009, 79 (24), 245326 10.1103/PhysRevB.79.245326.

[ref17] Lane P. A.; Cunningham P. D.; Melinger J. S.; Esenturk O.; Heilweil E. J. Hot Photocarrier Dynamics in Organic Solar Cells. Nat. Commun. 2015, 6, 7558 10.1038/ncomms8558. 26179323

[ref18] Bässler H. Charge Transport in Disordered Organic Photoconductors a Monte Carlo Simulation Study. Phys. Status Solidi B 1993, 175 (1), 15–56. 10.1002/pssb.2221750102.

[ref19] Melianas A.; Etzold F.; Savenije T. J.; Laquai F.; Inganäs O.; Kemerink M. Photo-Generated Carriers Lose Energy during Extraction from Polymer-Fullerene Solar Cells. Nat. Commun. 2015, 6, 8778 10.1038/ncomms9778. 26537357 PMC4659933

[ref20] Melianas A.; Pranculis V.; Xia Y.; Felekidis N.; Inganäs O.; Gulbinas V.; Kemerink M. Photogenerated Carrier Mobility Significantly Exceeds Injected Carrier Mobility in Organic Solar Cells. Adv. Energy Mater. 2017, 7 (9), 1602143 10.1002/aenm.201602143.

[ref21] Brigeman A. N.; Fusella M. A.; Rand B. P.; Giebink N. C. Nonthermal Site Occupation at the Donor-Acceptor Interface of Organic Solar Cells. Phys. Rev. Appl. 2018, 10, 034034 10.1103/PhysRevApplied.10.034034.

[ref22] Melianas A.; Felekidis N.; Puttisong Y.; Meskers S. C. J.; Inganäs O.; Chen W. M.; Kemerink M. Nonequilibrium Site Distribution Governs Charge-Transfer Electroluminescence at Disordered Organic Heterointerfaces. Proc. Natl. Acad. Sci. U. S. A. 2019, 116 (47), 23416–23425. 10.1073/pnas.1908776116. 31690666 PMC6876215

[ref23] Kirchartz T.; Nelson J.; Rau U. Reciprocity between Charge Injection and Extraction and Its Influence on the Interpretation of Electroluminescence Spectra in Organic Solar Cells. Phys. Rev. Appl. 2016, 5 (5), 054003 10.1103/PhysRevApplied.5.054003.

[ref24] Wilken S.; Upreti T.; Melianas A.; Dahlström S.; Persson G.; Olsson E.; Österbacka R.; Kemerink M. Experimentally Calibrated Kinetic Monte Carlo Model Reproduces Organic Solar Cell Current–Voltage Curve. Solar RRL 2020, 4 (6), 2000029 10.1002/solr.202000029.

[ref25] Pasveer W. F.; Cottaar J.; Tanase C.; Coehoorn R.; Bobbert P. A.; Blom P. W. M.; de Leeuw D. M.; Michels M. A. J. Unified Description of Charge-Carrier Mobilities in Disordered Semiconducting Polymers. Phys. Rev. Lett. 2005, 94, 206601 10.1103/PhysRevLett.94.206601. 16090265

[ref26] Burkhard G. F.; Hoke E. T.; McGehee M. D. Accounting for Interference, Scattering, and Electrode Absorption to Make Accurate Internal Quantum Efficiency Measurements in Organic and Other Thin Solar Cells. Adv. Mater. 2010, 22 (30), 3293–3297. 10.1002/adma.201000883. 20517871

[ref27] Bartesaghi D.; Pérez I. d. C.; Kniepert J.; Roland S.; Turbiez M.; Neher D.; Koster L. J. A. Competition between Recombination and Extraction of Free Charges Determines the Fill Factor of Organic Solar Cells. Nat. Commun. 2015, 6, 7083 10.1038/ncomms8083. 25947637 PMC4432638

[ref28] Neher D.; Kniepert J.; Elimelech A.; Koster L. J. A. A New Figure of Merit for Organic Solar Cells with Transport-Limited Photocurrents. Sci. Rep. 2016, 6 (1), 24861 10.1038/srep24861. 27112905 PMC4845057

[ref29] Wilken S.; Sandberg O. J.; Scheunemann D.; Österbacka R. Watching Space Charge Build Up in an Organic Solar Cell. Solar RRL 2020, 4 (3), 1900505 10.1002/solr.201900505.

[ref30] Mihailetchi V. D.; Wildeman J.; Blom P. W. M. Space-Charge Limited Photocurrent. Phys. Rev. Lett. 2005, 94 (12), 126602 10.1103/PhysRevLett.94.126602. 15903944

[ref31] Felekidis N.; Melianas A.; Kemerink M. The Role of Delocalization and Excess Energy in the Quantum Efficiency of Organic Solar Cells and the Validity of Optical Reciprocity Relations. J. Phys. Chem. Lett. 2020, 11 (9), 3563–3570. 10.1021/acs.jpclett.0c00945. 32301322

[ref32] Ross R. T.; Nozik A. J. Efficiency of Hot-carrier Solar Energy Converters. J. Appl. Phys. 1982, 53 (5), 3813–3818. 10.1063/1.331124.

[ref33] Würfel P. Solar Energy Conversion with Hot Electrons from Impact Ionisation. Sol. Energy Mater. Sol. Cells 1997, 46 (1), 43–52. 10.1016/S0927-0248(96)00092-X.

[ref34] Kahmann S.; Loi M. A. Hot Carrier Solar Cells and the Potential of Perovskites for Breaking the Shockley–Queisser Limit. J. Mater. Chem. C 2019, 7 (9), 2471–2486. 10.1039/C8TC04641G.

[ref35] Mihailetchi V. D.; Koster L. J. A.; Hummelen J. C.; Blom P. W. M. Photocurrent Generation in Polymer-Fullerene Bulk Heterojunctions. Phys. Rev. Lett. 2004, 93 (21), 216601 10.1103/PhysRevLett.93.216601. 15601044

[ref36] Roland S.; Kniepert J.; Love J. A.; Negi V.; Liu F.; Bobbert P.; Melianas A.; Kemerink M.; Hofacker A.; Neher D. Equilibrated Charge Carrier Populations Govern Steady-State Nongeminate Recombination in Disordered Organic Solar Cells. J. Phys. Chem. Lett. 2019, 10 (6), 1374–1381. 10.1021/acs.jpclett.9b00516. 30829040

[ref37] Marianer S.; Shklovskii B. I. Effective Temperature of Hopping Electrons in a Strong Electric Field. Phys. Rev. B 1992, 46 (20), 13100–13103. 10.1103/PhysRevB.46.13100. 10003349

[ref38] Yuan J.; Zhang Y.; Zhou L.; Zhang G.; Yip H.-L.; Lau T.-K.; Lu X.; Zhu C.; Peng H.; Johnson P. A.; Leclerc M.; Cao Y.; Ulanski J.; Li Y.; Zou Y. Single-Junction Organic Solar Cell with over 15% Efficiency Using Fused-Ring Acceptor with Electron-Deficient Core. Joule 2019, 3 (4), 1140–1151. 10.1016/j.joule.2019.01.004.

[ref39] Upreti T.; Wang Y.; Zhang H.; Scheunemann D.; Gao F.; Kemerink M. Experimentally Validated Hopping-Transport Model for Energetically Disordered Organic Semiconductors. Phys. Rev. Appl. 2019, 12 (6), 064039 10.1103/PhysRevApplied.12.064039.

[ref40] Vandewal K.; Albrecht S.; Hoke E. T.; Graham K. R.; Widmer J.; Douglas J. D.; Schubert M.; Mateker W. R.; Bloking J. T.; Burkhard G. F.; Sellinger A.; Fréchet J. M. J.; Amassian A.; Riede M. K.; McGehee M. D.; Neher D.; Salleo A. Efficient Charge Generation by Relaxed Charge-Transfer States at Organic Interfaces. Nat. Mater. 2014, 13 (1), 63–68. 10.1038/nmat3807. 24240240

[ref41] Qian D.; Zheng Z.; Yao H.; Tress W.; Hopper T. R.; Chen S.; Li S.; Liu J.; Chen S.; Zhang J.; Liu X.-K.; Gao B.; Ouyang L.; Jin Y.; Pozina G.; Buyanova I. A.; Chen W. M.; Inganäs O.; Coropceanu V.; Bredas J.-L.; Yan H.; Hou J.; Zhang F.; Bakulin A. A.; Gao F. Design Rules for Minimizing Voltage Losses in High-Efficiency Organic Solar Cells. Nat. Mater. 2018, 17 (8), 703–709. 10.1038/s41563-018-0128-z. 30013057

[ref42] Mikhnenko O. V.; Blom P. W. M.; Nguyen T.-Q. Exciton Diffusion in Organic Semiconductors. Energy Environ. Sci. 2015, 8 (7), 1867–1888. 10.1039/C5EE00925A.

[ref43] Burke T. M.; McGehee M. D. How High Local Charge Carrier Mobility and an Energy Cascade in a Three-Phase Bulk Heterojunction Enable >90% Quantum Efficiency. Adv. Mater. 2014, 26 (12), 1923–1928. 10.1002/adma.201304241. 24375640

[ref44] Andersson O.; Kemerink M. Enhancing Open-Circuit Voltage in Gradient Organic Solar Cells by Rectifying Thermalization Losses. Solar RRL 2020, 4 (12), 2000400 10.1002/solr.202000400.

