# Supplementary material for: Slow Relaxation of Photogenerated Charge Carriers Boosts Open-Circuit Voltage of Organic Solar Cells
Source: J Phys Chem Lett. 2021 Oct 5;12(40):9874–81. doi: 10.1021/acs.jpclett.1c02235 (PMC8521526; doi:10.1021/acs.jpclett.1c02235)
Supplement: Supplementary file 1 — jz1c02235_si_001.pdf [file jz1c02235_si_001.pdf]

Supporting Information: Slow Relaxation of Photogenerated Charge Carriers Boosts Open-Circuit Voltage of Organic Solar Cells

Tanvi Upreti,[†,‡] Sebastian Wilken,[†,¶] Huotian Zhang,[§] and Martijn Kemerink[*,†,‡]

†*Complex Materials and Devices, Department of Physics, Chemistry and Biology (IFM), Linköping University, 581 83 Linköping, Sweden*

‡*Centre for Advanced Materials, Heidelberg University, Im Neuenheimer Feld 225, 69120 Heidelberg, Germany*

¶*Physics, Faculty of Science and Engineering, Åbo Akademi University, Porthansgatan 3, 20500 Turku, Finland*

§*Biomolecular and Organic Electronics, Department of Physics, Chemistry and Biology (IFM), Linköping University, 581 83 Linköping, Sweden*

* E-mail: martijn.kemerink@cam.uni-heidelberg.de

Contents

1 Experimental Details **S-3**

2 Details of the Numerical Models **S-6**

2.1 Kinetic Monte Carlo Model S-6

2.2 Drift–Diffusion Model S-11

2.3 Optical Modeling S-12

3 Reciprocity Analysis of KMC Simulations **S-14**

4 Transient Energetics of Photogenerated Charges **S-19**

5 Current–Voltage Curves at Large Reverse Bias **S-24**

6 Temperature Dependent Drift–Diffusion Simulations **S-25**

7 Temperature Dependence for a Thick TQ1:PC_{71}BM Device **S-27**

8 Role of Energetic Disorder **S-28**

9 Yields for Drift–Diffusion **S-30**

References **S-32**

1 Experimental Details

Materials The chemical structures of the photoactive materials used in this study are shown in Figure S1. The poly[[2,3-bis(3-octyloxyphenyl)-5,8-quinoxalinediyl]-2,5-thiophenediyl] (TQ1) polymer was synthesized as described previously.[S1] [6,6]-phenyl-C_{71}-butyric acid methyl ester (PC$_{71}$BM) was purchased from 1-Material. Poly[(2,6-(4,8-bis(5-(2-ethylhexyl-3-fluoro) thiophen-2-yl)-benzo[1,2-b:4,5-b′]dithiophene))-alt-(5,5-(1′,3′-di-2-thienyl-5′,7′-bis (2-ethylhexyl)benzo[1′,2′-c:4′,5′c′]dithiophene-4,8-dione (PM6) was purchased from Solarmer Materials. 2,2′-((2Z,2′Z)-((12,13-bis(2-ethylhexyl)-3,9-diundecyl-12,13-dihydro-[1,2,5]thiadiazolo[3,4-e]thieno[2,″30″:4′,5′]thieno[2′,3′:4,5]pyrrolo[3,2-g]thieno[2′,3′:4,5]thieno[3,2-b]indole-2,10-diyl)bis(methanylylidene))bis(5,6-difluoro-3-oxo-2,3-dihydro-1H-indene-2,1-diylidene))dimalononitrile(Y6) was synthesized according to literature.[S2]

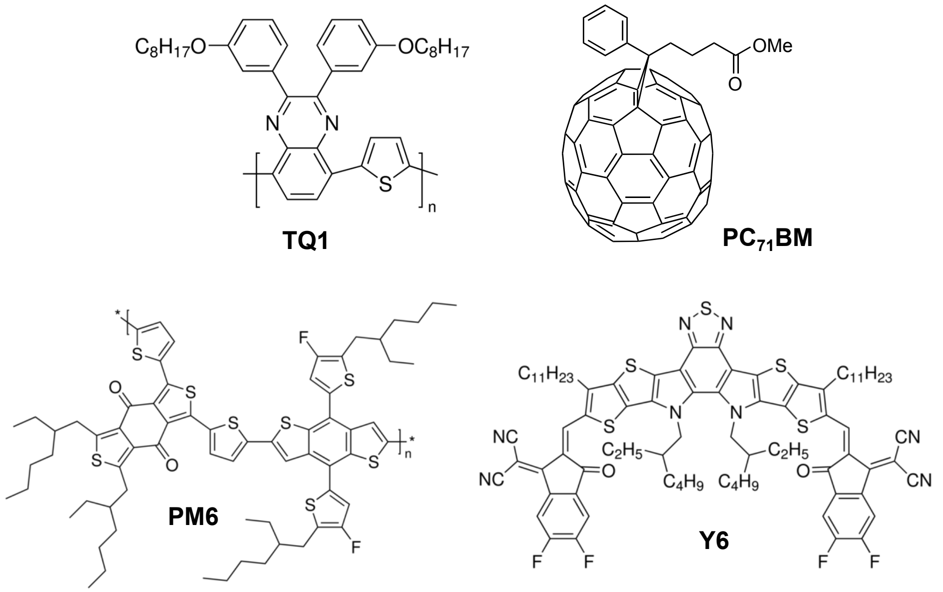

Figure S1: Chemical structures of the photoactive materials used in this study.

Device fabrication TQ1:PC$_{71}$BM solar cells were fabricated with the structure indium tin oxide (ITO)/poly(3,4-ethylenedioxythiophene) polystyrene sulfonate (PEDOT:PSS)/active layer/LiF/Al. The PEDOT:PSS aqueous solution (Baytron P VP AI 4083) was spin-coated

on cleaned ITO-covered glass substrates at 3000 rpm for 15 s, followed by annealing at 150 °C for 10 min in air, to form a 30 nm film. The active layer was spin-coated at 500 rpm for 60 s from a 1:2.5 (w/w) blend solution of TQ1 and $PC_{71}BM$ in chlorobenzene. The concentration of the solution was varied from 20 to 100 mg ml^{-1} to yield an active-layer thickness of 70–310 nm. Finally, the top electrode of LiF (0.6 nm) and Al (90 nm) was thermally evaporated under high vacuum. PM6:Y6 solar cells were fabricated with the structure ITO/PEDOT:PSS/active layer/PFN-Br/Al. The active layer was spin-coated at 2500–6000 rpm from a 1:1.2 (w/w) blend solution of PM6 and Y6 in chloroform with 0.5% 1-chloronaphthalene as an additive. Subsequently, the electron-extracting layer of PFN-Br (Solarmer Materials) was spin-coated from a 0.5 mg ml^{-1} solution in methanol. Finally, the top electrode was evaporated under high vacuum.

Characterization Film thicknesses were determined using a Dektak 6M stylus profilometer. The thicknesses of the devices were averaged over 4 samples each with 4 pixels. Room temperature current–voltage curves were recorded with a Keithley 2400 source measure unit under standard AM1.5G illumination (100 mW cm^{-2}) using an Oriel LSH-7320 solar simulator. For temperature-dependent measurements, TQ1:$PC_{71}BM$ devices were mounted in a liquid-nitrogen cryostat controlled by a Lake Shore 330 temperature controller. The assembly had a built-in solar simulator with a Xe-arc lamp which was calibrated for the spectral mismatch. PM6:Y6 devices were tested in a closed cycle helium cryostat (Advanced Research Systems) and illuminated with a 532 nm laser (Thorlabs). A Keithley 2400 Source Meter provided the voltage and measured current for both the systems. Temperature-dependent measurements were averaged over cooling and heating sweeps to account for minor time dependencies. The monochromatic excitation used for the PM6:Y6 devices is unproblematic for the current study that focusses on hot-carrier (distribution) effects. Since hot-carrier phenomena are based on the presence of excess photon energy, the condition for these to occur is that the difference between the photon energy and the energy gap of the absorber

is significant. For the 532 nm (2.33 eV) laser, the condition is easily met for both PM6 and Y6 that have an optical energy gap around 600 and 900 nm, respectively.[S2] In addition the 'hotness' of the charge carrier populations stems to a large degree from the energy gained upon charge transfer at the donor/acceptor interface, which happens as long as one does not selectively excite deep in the CT state.[S3]

2 Details of the Numerical Models

2.1 Kinetic Monte Carlo Model

The kinetic Monte Carlo (KMC) model is implemented on a simple cubic grid such that the nearest neighbor hopping distance a_{NN} equals the lattice constant and relates to the total site density N_0 as $a_{\mathrm{NN}} = N_0^{-1/3}$. While the code allows to consider hopping to different numbers of neighbor sites, we used strict nearest neighbor hopping here to warrant consistency with earlier work. Below the model and its parameters are explained in detail.

Hopping Rates We use the Miller-Abrahams expression to quantify, with the least number of parameters, the nearest-neighbor hopping rate of a charge carrier from an initial state i with energy E_i to a final state f with energy E_f as

$$\nu_{ij} = \nu_0 \exp(-\alpha r_{if}) \begin{cases} \exp\left(-\frac{E_f - E_i \pm q\vec{r}_{if}\cdot\vec{F} + \Delta E_C}{kT}\right) & \Delta E > 0 \\ 1 & \Delta E \leq 0 \end{cases} \tag{S1}$$

Here, $\vec{F}$ is the external electric field, $\vec{r}_{if}$ the vector connecting initial and final sites, ν_0 the attempt-to-hop frequency, and q the positive elementary charge. The + (−) sign refers to electron (hole) hopping. In the configuration used (strict nearest neighbor hopping), the localization length α is unimportant and the first exponential term of Equation (S1) was implicitly included in ν_0, that is, the rate of downward nearest-neighbor hops. In the context of this work, it is important that including non-nearest neighbors as final sites has a similar effect on the relaxation rate as the corresponding mobility increase by an increase in ν_0. That is, increasing the number of neighbors while keeping the mobility constant does not significantly affect the thermalization process.

Energetics The term ΔE_C is the change in Coulomb energy and is calculated by explicit evaluation of the interaction of the moving charge with (a) all other charges in the simulated

device and (b) their image charges, as well as of the interaction of the image charges of the moving particle with (c) the particle itself and (d) all other particles. Image charges arise when metallic contacts are present; the number of image charges accounted for in the simulations is increased till the resulting effective Coulomb potential no longer changes. In order to avoid divergences at zero separation, the Coulomb interaction between a pair of (unlike) charges, $E_C = -q/(4\pi\varepsilon_0\varepsilon_r r_{\mathrm{eh}})$ with $\varepsilon_0\varepsilon_r$ the dielectric constant ($\varepsilon_r = 3.6$) and r_{eh} the electron-hole distance, is truncated at minus the approximate exciton binding energy of $E_b^{\mathrm{ex}} = 0.5\,\mathrm{eV}$. The single-particle site energies E_i are drawn from a Gaussian distribution function

$$g(E) = \frac{1}{\sqrt{2\pi\sigma^2}} \exp\left[-\frac{(E - E_0)^2}{2\sigma^2}\right] \tag{S2}$$

with E_0 the mean energy and σ the broadening of the total density of states (DOS) N_0. The HOMO and LUMO energy of a single site are assumed to be uncorrelated.

Morphology In previous works, we used an effective hopping medium with (different) electron and hole hopping parameters ν_0 and σ that correspond to the donor HOMO and acceptor LUMO levels, respectively. The driving force for charge transfer is then implemented via an on-site electron-hole repulsion with a magnitude that equals the LUMO level offset $\Delta E_{\mathrm{LUMO}} = \Delta E^D_{\mathrm{LUMO}} - \Delta E^A_{\mathrm{LUMO}}$ between donor and acceptor. Here, and in Ref. S4, we implemented a simplified phase separated morphology for the TQ1:PC_{71}BM system as columnar inclusions ($7 \times 7\,\mathrm{sites}^2$) in an columnar unit cell ($10 \times 10\,\mathrm{sites}^2$) where the column axis runs in the current direction (z). Inclusions were assumed to consist of pure PC_{71}BM with a 0.2 eV lower-lying LUMO compared to the mixed phase; all other properties were left unchanged to keep the number of unknown parameters at a minimum. We did not consider pure TQ1 domains, as our previous electron microscopy experiments do not provide any evidence for them.[S4] By lack of specific morphological information, the same morphology was used for the PM6:Y6 system.

Excitons Spatially direct excitons, formed by an electron and a hole on the same site, can recombine with rate ν_{ex}. Similarly, when sitting on neighboring sites, they form a CT complex that can recombine with rate ν_{CT}. This implies that mono- and bimolecular recombination are treated on equal footing, as recombination rates of exciton and CT species do not depend on the history of the constituent charges. Exciton diffusion by the Förster resonant energy transfer (FRET) mechanism is explicitly accounted for. The transition rate is evaluated as

$$\nu_{if}^{F} = \nu_{\mathrm{ex}} \left(\frac{R_0}{r_{if}} \right)^6 \Theta\left(E_i^{\mathrm{ex}} - E_f^{\mathrm{ex}} \right) \tag{S3}$$

where R_0 is the Förster radius, ν_{ex} the radiative exciton decay rate, Θ the Heaviside step function, and E_i^{ex}, E_f^{ex} the exciton energies $E_{i/f}^{\mathrm{ex}} = E_{i/f}^{\mathrm{LUMO}} - E_{i/f}^{\mathrm{HOMO}} - E_b^{\mathrm{ex}}$ at the initial and final sites. Dexter-type exciton diffusion is implicitly accounted for as a double charge hopping process.

Kinetics The waiting time before an event (hop or recombination) occurs is calculated as

$$\tau = -\frac{\ln(r)}{\Sigma_\nu} \tag{S4}$$

where r is a random number drawn from a homogeneous distribution between 0 and 1 and Σ_ν is the sum of the rates of all possible events. The event that occurs after τ is selected randomly, using the rates of all possible events as weight factors. Energies, rates, and waiting time are recalculated after each event.

Contacts and Boundary Conditions Periodic boundary conditions in the x,y-directions were applied for both charge motion and (image and direct) Coulomb interactions; contacts laying in the z-plane are included unless stated otherwise and were implemented as hopping contacts. We mitigated the 'small barrier' problem (carriers oscillating across the contact interface at large computational cost) by only allowing for a transfer if the number of charges

Table S1: Overview of the parameters used for the kinetic Monte Carlo simulation of TQ1:PC$_{71}$BM and PM6:Y6 solar cells. HOMO and LUMO refer to the orbital energies of the effective medium.

	TQ1:PC$_{71}$BM	PM6:Y6	
Parameter [unit]	Value[S4]	Value	Literature value
Nearest neighbor distance, a_{NN} [nm]	1.8	1.8	1.8[S6]
LUMO acceptor, E^{A}_{LUMO} [eV]	3.8	4.0	4.0[S7]
HOMO donor, E^{D}_{HOMO} [eV]	5.2	5.44	5.48[S7]
Attempt-to-hop frequency electrons, $\nu_{0,e}$ [s^{-1}]	1×10^{11}	1×10^{11}	1.6×10^{10}[S8]
Attempt-to-hop frequency holes, $\nu_{0,h}$ [s^{-1}]	1×10^{10}	1×10^{11}	1.6×10^{9}[S8]
Energetic disorder electrons, σ_e [meV]	75	70	68[S8]
Energetic disorder holes, σ_h [meV]	75	70	89[S8]
Inverse exciton lifetime, ν_{ex} [s^{-1}]	1×10^{9}	1×10^{9}	
Inverse CT state lifetime, ν_{CT} [s^{-1}]	3×10^{7}	3×10^{7}	
Injection barrier height [eV]	0.2	0.2	

next to the contact interface deviates from its equilibrium value, which is calculated as a Fermi-integral over the actual DOS in the first organic layer next to the contact:[S5]

$$n_{\mathrm{cont}} = \int_{-\infty}^{\infty} \frac{g(E)}{1 + \exp(E/kT)} \, \mathrm{d}E \tag{S5}$$

Injection and extraction are modeled as hopping events with an attempt frequency $\nu_{0,\mathrm{cont}}$ of the same order as for the transport of the faster carrier in the semiconductor. We explicitly checked that this procedure does not limit charge collection or extraction at the contacts for the given parameters and voltages. We also checked that V_{OC} is not significantly affected by the use of these 'buffered' hopping contacts by running a single J–V point with non-buffered hopping at V_{OC}. Both the cathode and anode were considered nonselective; hence, possible losses due to diffusion of carriers into the 'wrong' contact are implicitly accounted for.

Input Parameters For the TQ1:PC$_{71}$BM system, the parameters in Table S1 above are taken from our earlier work,[S4] where we also motivate the choice of equal values for the energetic disorder for electrons and holes. In short, the choice for symmetric disorder values and attempt-to-hop frequencies that differ by an order of magnitude roughly maintains

the right, experimentally determined, mobility values and ratio while keeping calculation times manageable. A similar reasoning was used for setting the parameters for the PM6:Y6 system, for which much less KMC-relevant parameters have been published previously and the uncertainty therefore is larger. As for the TQ1:$PC_{71}BM$ system, we symmetrized the hopping parameters for numerical reasons. The changes in the hole parameters, which were needed to reproduce the experimental fill factor, actually lead to a reduced effect of disorder in the form of faster thermalization as compared to the values in the rightmost column. From our experience, rather substantial parameter fluctuations for different experiments performed on different batches of nominally the same material, are unfortunately not uncommon.

Simulation Procedures The KMC model was calibrated for each material system to describe the J–V curve of a *single* device thickness at a given temperature, as described in Ref. S4, after which all parameters were kept constant. In the calibration step, the energy levels were adjusted to match V_{OC}, by calculating a J–V curve using literature values for the acceptor LUMO and donor HOMO levels and subsequently adjusting one of those to make the calculated V_{OC} equal to the measured value. Since all other parameters are kept constant, V_{OC} is linear in the effective band gap and this procedure converges in a single iteration. Other parameters like the injection barrier heights and optical constants were determined by independent experiments. The fill factor was not explicitly calibrated. The calculated dependencies on thickness and temperature are therefore model predictions and not fits. That the model captures the (V_{OC}) behavior that is relevant to the present argument is not something the model was 'tweaked' to do. Instead, it is a consequence of the physics that is included. KMC simulations were performed on boxes containing 40^3 sites, which for a nearest neighbor distance of 1.8 nm corresponds to 72^3 nm^3. Averages over multiple random configurations of the site energies were taken until the resulting error bars were sufficiently small, i.e., of the size of the symbols used in the figures. Using the described methodology, full J–V curves can be simulated with sufficient accuracy for direct comparison with exper-

iments. Unfortunately, both the calculation times and the numerical uncertainty tend to go up around open circuit, which precludes meaningful statements about the illumination intensity dependence of V_{OC}. Especially at sub-1-sun intensities, which is the regime at which experiments can be done without heating and degradation effects setting in, the numerical noise due to the presence of Ohmic contacts quickly overwhelms any photoinduced signals despite the use of the 'buffered' contacts described above.

2.2 Drift–Diffusion Model

The drift–diffusion (DD) model solves a set of coupled differential equations, namely the Poisson equation and the continuity equations for electrons and holes, using the one-dimensional Scharfetter–Gummel discretisation.[S9] Charge recombination was treated in terms of a bimolecular rate equation, $R = k_2 np$, where k_2 is the recombination rate constant and n and p the density of electrons and holes. Further details of the specific implementation can be found in Ref. S6. To take into account the (Gaussian) disorder and to make the DD model as comparable as possible to the KMC model, three measures were implemented:

1. The generalized Einstein equation[S10] was used for the relation between the diffusion coefficient and the mobility.

2. As boundary conditions at the contacts, the interfacial charge density was used, which was explicitly calculated as the integral over the Gaussian DOS multiplied with the Fermi–Dirac distribution, see Eq. (S5). That means that the same boundary condition is used as for the 'buffered' contacts in the KMC model.[S5,S11] For non-Ohmic contacts, defined as having an electric field F_C at the semiconductor/metal interface that directs a drift current away from the metal, the injection barrier is lowered by the image potential as:[S5]

$$\Delta' = \Delta - q\sqrt{\frac{q|F_C|}{4\pi\varepsilon_0\varepsilon_r}} \tag{S6}$$

   For F_C directing a drift current towards the metal (Ohmic contacts) the full injection

Table S2: Overview of the parameters used for the drift–diffusion simulation of TQ1:PC$_{71}$BM and PM6:Y6 solar cells.

Parameter [unit]	Value	
	TQ1:PC$_{71}$BM	PM6:Y6
Effective energy gap [eV]	1.4	1.44
Nearest neighbor distance, a_{NN} [nm]	1.8	1.8
Attempt-to-hop frequency electrons, $\nu_{0,e}$ [s^{-1}]	1×10^{11}	1×10^{11}
Attempt-to-hop frequency holes, $\nu_{0,h}$ [s^{-1}]	1×10^{10}	1×10^{11}
Energetic disorder [meV]	75	70
Recombination rate constant, k_2 [m^{3}s^{-1}]	2×10^{-17}	2×10^{-17}
Injection barrier height [eV]	0.2	0.2

barrier Δ is used.

3. The mobility functional for the extended Gaussian disorder model by Pasveer et al.[S12] was applied to translate the same set of hopping parameters (a_{NN}, ν_0, σ) as used in the KMC model into a quasi-equilibrium mobility. It was checked that the KMC model reproduces the mobility values from Pasveer et al. Hence, for thermalized charge carriers, KMC and DD use the same mobility values; evidently, since in DD all charge carriers are assumed to be thermalized, the in KMC naturally included effect that nonthermalized charge are more mobile is absent in DD.

All relevant input parameters for the DD model are given in Table S2. Note that all parameters are equal to the corresponding numbers used in the KMC model (Table S1) with the exception of the recombination rate constant that is not an independent parameter in KMC and that was taken from literature.[S13] The effective energy gap is calculated as the difference between the donor HOMO energy and the acceptor LUMO energy.

2.3 Optical Modeling

To take into account the spatial profile of the optical generation rate and its variation with thickness, the spatial distribution of photocreated singlet excitons was calculated for the TQ1:PC$_{71}$BM system using the transfer-matrix approach as described previously.[S14]

The used optical constants were determined by spectroscopic ellipsometry and are shown in Figure S2. Since no systematic thickness-dependent study was performed for the PM6:Y6 system, optical modeling was not needed, and a constant generation rate was used.

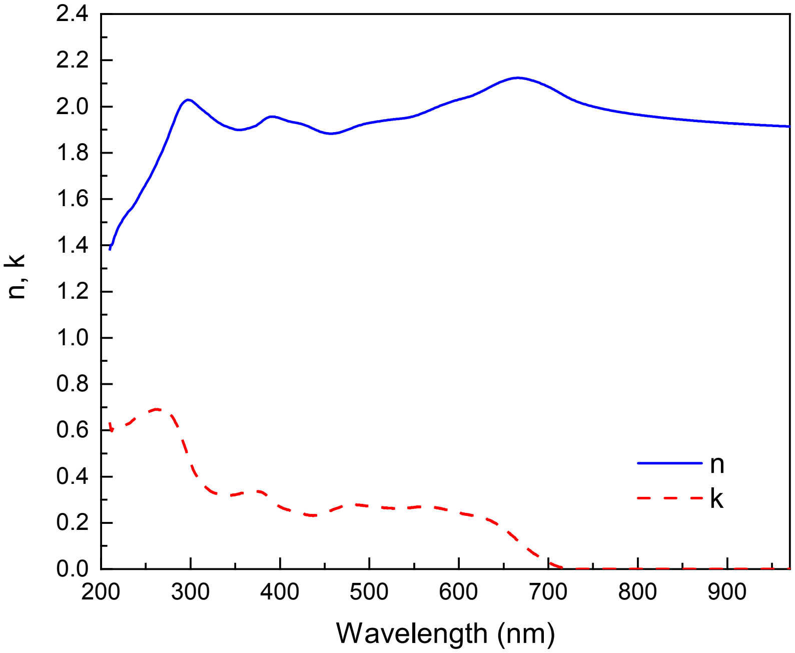

Figure S2: Optical constants for TQ1:$PC_{71}BM$ as used in the transfer matrix modeling.

3 Reciprocity Analysis of KMC Simulations

In our reciprocity analysis we considered an extended version of Eqs. 1 and 2 in the main text,

$$V_{\mathrm{OC}} = \frac{kT}{q} \ln\left(\frac{J_{\mathrm{gen}}}{J_0} + 1\right) + \frac{kT}{q} \ln(\mathrm{EQE}_{\mathrm{EL}}) + \frac{kT}{q} \ln\left(\frac{F_{\mathrm{coll}}}{F_{\mathrm{inj}}}\right) \tag{S7}$$

Here, the first term on the right-hand side is the radiative limit for V_{OC}. The second term accounts for losses due to nonradiative recombination. The third term accounts for differences in collection efficiency (during photovoltaic operation under illumination) and injection efficiency (during operation as light emitting device in the dark), as derived by Kirchartz et al.[S15] The value for $\mathrm{EQE}_{\mathrm{EL}}$ can be determined from KMC simulations by running the same device as light emitting diode, i.e., in the dark under a forward bias corresponding to V_{OC}, and dividing the integrated recombination current by the total injection current,[S3]

$$\mathrm{EQE}_{\mathrm{EL}} = \frac{J_{\mathrm{EL}}}{J_{\mathrm{inj}}} \tag{S8}$$

For the used parameters and morphology, a value of $\mathrm{EQE}_{\mathrm{EL}} = 0.14$ was found for TQ1:PC_{71}BM. Likewise, the values for F_{inj} and F_{coll} can be obtained from KMC simulations at $V = V_{\mathrm{OC}}$ using

$$F_{\mathrm{inj}} = \frac{1}{d} \int_0^d \frac{n(x,V)p(x,V) - n_i^2}{n_i^2(\exp(qV/kT) - 1)} \, \mathrm{d}x \tag{S9}$$

where n and p are the electron and hole densities, d the active-layer thickness and n_i the intrinsic (thermal) charge density corresponding to the effective energy gap, $n_i^2 = N_0^2 \exp(-E_{\mathrm{gap}}^{\mathrm{eff}}/kT)$ with the site density $N_0 = a_{\mathrm{NN}}^{-3}$, and

$$F_{\mathrm{coll}} = \frac{1}{d} \int_0^d f_c(x,V) \, \mathrm{d}x \tag{S10}$$

Where, $f_c(x,V)$ is the collection probability of charges photogenerated at a position x in the device. The spatially averaged values for electrons and holes are the same, and equal

to the ratio of the photocurrent (light minus dark current) and the maximum generated photocurrent J_{gen} that is either determined by optical modeling (see Section 2.3 above) or as $J_{\mathrm{gen}} = qG_{\mathrm{av}}d$ with G_{av} the average exciton generation rate.[S15] Hence,

$$F_{\mathrm{coll}} = \frac{J_{\mathrm{photo}}}{J_{\mathrm{gen}}} \tag{S11}$$

For the TQ1:$PC_{71}BM$ system, for which this analysis is performed, F_{inj} and F_{coll} were found to be equal, with a value $F_{\mathrm{inj}} = F_{\mathrm{coll}} = 0.32$, meaning that the last term in Eq. (S7) actually becomes zero.

Finally, the reverse dark saturation current can be obtained by integrating Eq. (2) of the main text as

$$J_0 = q\int \mathrm{EQE}_{\mathrm{PV}}(E)\phi_{\mathrm{BB}}(E)\,\mathrm{d}E \tag{S12}$$

for which we use

$$\mathrm{EQE}_{\mathrm{PV}}(E) = \mathrm{IQE}_{\mathrm{PV}}(E)\phi_{\mathrm{abs}}(E) \tag{S13}$$

with $\mathrm{IQE}_{\mathrm{PV}}$ the internal quantum efficiency for photovoltaic operation that is set to unity unless stated otherwise[S3] and ϕ_{abs} the absorption spectrum. Due to the steepness of the black body spectrum ϕ_{BB},

$$\phi_{\mathrm{BB}}(E) = \frac{2\pi E^2}{h^3c^2}\frac{1}{\exp(E/kT)-1} \quad [\mathrm{m}^{-2}\mathrm{s}^{-1}\mathrm{J}^{-1}], \tag{S14}$$

only the energetically lowest parts of the CT and S1 contributions to ϕ_{abs} are important. We write ϕ_{abs} as

$$\phi_{\mathrm{abs}}(E) = a\phi_{\mathrm{CT}}(E) + b\phi_{\mathrm{S1}}(E), \tag{S15}$$

where the (TQ1:$PC_{71}BM$) CT and (TQ1) singlet absorption spectra are calculated as convolutions of the relevant HOMO and LUMO levels as described before;[S3] their central energies are corrected for the Coulomb binding energies of the S1 (0.5 eV) and CT (0.22 eV) states.

The weight factors a and b are estimated as follows. Since TQ1 is a strong absorber, we take $b = 1$ at the absorption maximum, i.e., all photons impinging on the sample with the energy of the absorption maximum get absorbed. The factor a can then be estimated from

$$\frac{a}{b} = \frac{\nu_{\mathrm{CT}}}{\nu_{\mathrm{S1}}} \frac{n_{s,\mathrm{CT}}}{n_{s,\mathrm{S1}}} \tag{S16}$$

where $\nu_{\mathrm{S1}} = 1 \times 10^9\,\mathrm{s}^{-1}$ and $\nu_{\mathrm{CT}} = 3 \times 10^7\,\mathrm{s}^{-1}$ are the S1 and CT recombination rates as used in the model (see Table S1); these values have been calibrated to recombination transients.[S4] Equation (S16) makes the reasonable assumption that CT and S1 recombination are competing against the same or at least similar loss channels, such that their relative lifetimes reflect their relative oscillator strengths. The second term on the right-hand side of Eq. (S16) accounts for the fact that the number of absorption sites in the simulation box, n_s, is different for S1 and CT absorption. For the simplified morphology used here, a 10×10 unit cell with 7×7 inclusions of aggregated $\mathrm{PC_{71}BM}$, the lowest CT states are found at the interface between the mixed matrix and the $\mathrm{PC_{71}BM}$ inclusions, giving rise to a ratio $n_{s,\mathrm{CT}}/n_{s,\mathrm{S1}} = 28/51 \approx 0.55$.

The parameters listed in Table S1 give rise to the absorption spectrum in Figure S3, where also the black body spectrum is shown. Despite the simplifications made, the ratio of the S1 and CT absorption peaks of $\sim$0.017 that follows from Eq. (S16) is consistent with the experimentally observed value that falls in the range of 0.01 to 0.033, depending on whether on considers only the 0–0 transition, as done here, or the full vibronic progression.[S3] This suggests that the assumptions made are reasonable from a physical perspective. From Eqs. (S7)–(S16) we then obtain an equilibrium value of $V_{\mathrm{OC}} \approx 0.69\,\mathrm{V}$.

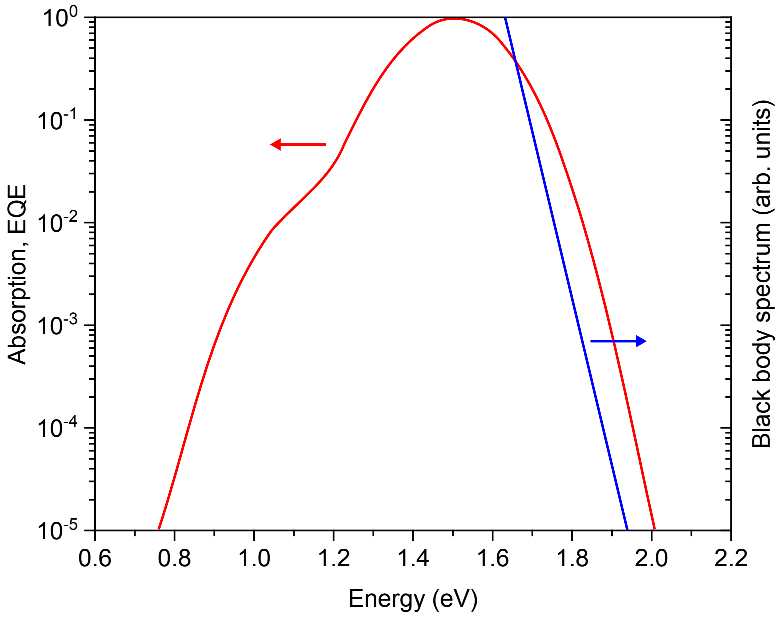

Figure S3: Absorption spectrum calculated from the parameters in Table S1 and Eqs. (S15) and (S16). The CT contribution is visible as a hump on the low-energy side of the spectrum. The main peak is the S1 contribution. The black body spectrum for $T = 300\,\mathrm{K}$, Eq. (S14), is shown in blue. The donor LUMO energy, which is not needed in the KMC simulations, is taken $E^{D}_{\mathrm{LUMO}} = 3.2\,\mathrm{eV}$. Since only the lowest part of the absorption spectrum is relevant for the calculation of J_0, Eqs. (S12) and (S13), the more or less flat continuum that is found in experiments at energies beyond the S1 peak is not shown.

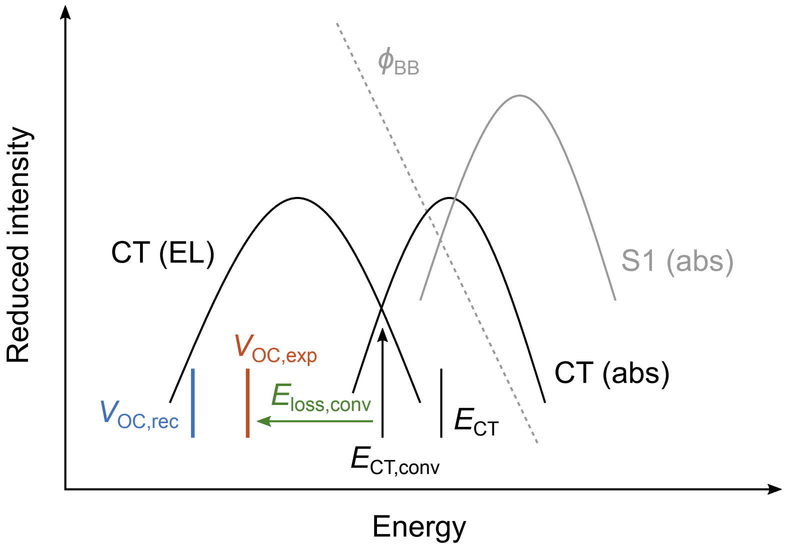

Figure S4: Sketch of the energetics and optical spectra. The vertical lines show the relative positions of characteristic energies with respect to the CT electroluminescence (EL) and absorption (abs) spectra and the red tail of the singlet (S1) absorption spectrum. The measured $V_{\mathrm{OC,exp}}$ is conventionally interpreted in terms of a loss energy w.r.t. the intersection of the CT EL and absorption spectra, labeled $E_{\mathrm{CT,conv}}$, that is referred to as the (relaxed) CT state. In our earlier work,[S16] we have shown that the actual relaxed CT energy lies at much lower energies (in fact, slightly below $V_{\mathrm{OC,exp}}$ for the TQ1:$PC_{71}BM$ system, not shown), and is therefore not a relevant point of reference. The central CT energy, labeled E_{CT}, lies above $E_{\mathrm{CT,conv}}$ but is not directly experimentally accessible. Assuming electron and hole populations are in thermodynamic equilibrium within their respective bands, as in the reciprocity analysis performed above, leads to a prediction for V_{OC} that underestimates the actual value by $\sim$0.2 eV, c.f. the blue line labeled $V_{\mathrm{OC,rec}}$. Using the same starting parameters in a KMC model, which makes no upfront assumptions about charge populations being in equilibrium, does reproduce the measured open-circuit value, as shown in this work.

4 Transient Energetics of Photogenerated Charges

From the KMC calculations, the energy distribution of photogenerated charge carriers can be traced as a function of time after photogeneration, i.e., the electron and hole energies are followed as a function of time after the exciton from which they originate was generated. This calculation is done under steady state conditions and is thus relevant to device operation. Similar results as shown in Figure S5 below were previously shown in Refs. S4,S17–S19 The results show that even under short-circuit conditions, charge thermalization does not complete before the charge carriers have been extracted. This can be seen from the fact that the mean electron and hole energies (solid lines, lower panel) have not reached the equilibrium energy (dashed lines) at the time where essentially all charges have left the device by either extraction or recombination. For the used parameters, this is around $t = 10^{-6}$ s and $t = 10^{-5}$ s for electrons and holes, respectively, see the upper panel.

At higher charge carrier densities, thermalization is no longer to the equilibrium energy but to the (quasi-)Fermi level, which would be an alternative interpretation of the data in Figure S5. However, to further highlight that thermalization is incomplete and does not stop at a (quasi-)Fermi level, which would be an indication of local equilibrium, we note that the difference between the mean electron and hole energies, indicated by the arrow in the bottom panel, saturates at around 1.15–1.20 eV, which is ~0.3 eV above the measured and calculated (by KMC) open-circuit voltage. The possibility that thermalization in Figure S5 might stop due to charges reaching a quasi-Fermi level can also be ruled out from the fact that this would require the quasi-Fermi levels to lie ~0.1 eV, i.e., less than 2σ, below the center of the band, which in turn would require charge densities that are orders of magnitude larger than typically observed in organic solar cells under open-circuit conditions. This corroborates the picture sketched in Fig. 3 of the main text, in which, at $V = V_{\mathrm{OC}}$, a nonthermalized photocurrent is counteracted by an injection current coming from the (thermalized) contacts.

One could expect signatures of the nonthermalized populations of the photogenerated

electrons and holes in plots of calculated densities occupied states vs. energy. Unfortunately, even after several days of computation at a single voltage point, the statistics are not enough to discern any meaningful high energy tails to, or to determine differences in effective temperature from. The reason is that the statistically fluctuating charge carrier population of charges diffusing in from the contacts overwhelms any fast moving photogenerated charges.

Interestingly, it is possible to get further confirmation for the existence of nonequilibrium currents from the energy resolved current distributions, see Figure S6 below. We attribute the oscillating shape of the current densities in the dark to the way these plots are extracted from the KMC calculations, as further explained in the caption. More interesting is the difference between the current distributions under illumination and in the dark (blue lines), which especially in the case of short-circuit conditions shows a clear negative (since $J_{SC} < 0$) peak at $\sim$5.15 eV, i.e., near to the center of the DOS, that lies well above both the maximum of the density of occupied states and the oscillating background current distribution. Interestingly, the center of this high-energy peak lies at roughly the same energy as the mean energy of the relaxing hole distribution (black line in the lower panel of Figure S5), which is consistent with the notion that the photocurrent is predominantly carried by nonthermalized holes. Under open-circuit conditions (dashed lines), there is no clear peak at higher energies, but instead there is an evident negative tail in the same region ($\sim$5.1 eV and upwards). Note that the total area under the curve is zero at open-circuit conditions under illumination.

On basis of the results in Figures S5 and S6 and the schematic Figure S4, one would expect a different position for the emission maximum between EL, which originates from 'equilibrated' charges coming from the contacts and PL, which would come from only partially thermalized photogenerated charges. This topic was addressed before in Ref. S3 (where also experimental data are shown in Fig. S9). In short, KMC simulations do show a minor effect of the expected sign. However, the magnitude is very small due to 'equilibrium' EL also coming from a nonthermal subset of sites,[S20] and is much smaller than the experimentally observed difference, which we attribute to sample inhomogeneity.[S3]

The area under the energy-resolved current curves reflects the corresponding net hole current density. All curves have been averaged over the full device thickness to reach sufficient statistics. We believe the oscillating shape in absence of illumination to be due to a combination of two factors. First, they reflect the presence of an Ohmic contact in which a positive diffusion current is preferentially injected into low-lying empty states and is compensated by a negative drift current that flows closer to the transport energy. Second, since the curves had to be averaged over the full device, spatial variations in charge and current distributions in combination with band bending may cause additional features. It is important that the implemented contacts do not act as charge pumps, i.e. in absence of illumination, J–V curves go through the origin.

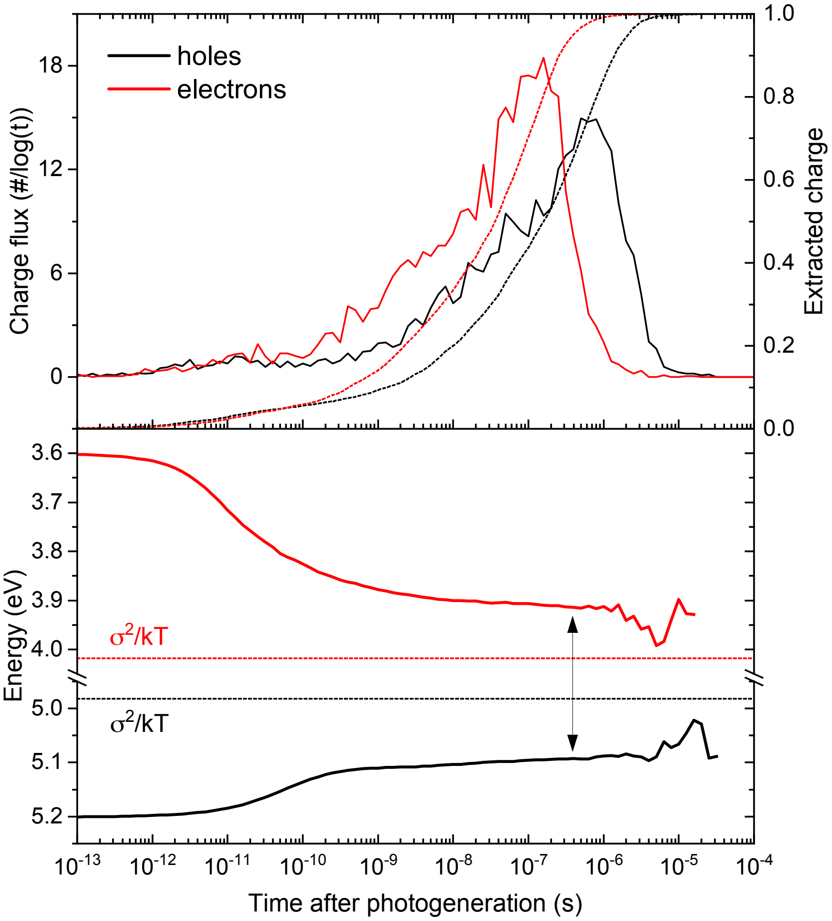

Figure S5: Energy loss after photoexcitation under open-circuit conditions for TQ1:PC$_{71}$BM. The top panel shows the extraction time distribution of photogenerated electrons (red) and holes (black) as solid lines, the corresponding integrated fraction of extracted charge is shown as dotted lines. The bottom panel shows the corresponding thermalization of photogenerated charges, the dotted horizontal lines indicate the equilibrium energies that lie σ^2/kT below (above) the LUMO (HOMO) energy. Note that the electrons are generated in the mixed TQ1:PC$_{71}$BM phase and loose an additional 0.2 eV upon transfer to the pure PC$_{71}$BM phase, which explains their larger apparent energy loss as compared to holes that remain in the mixed phase.

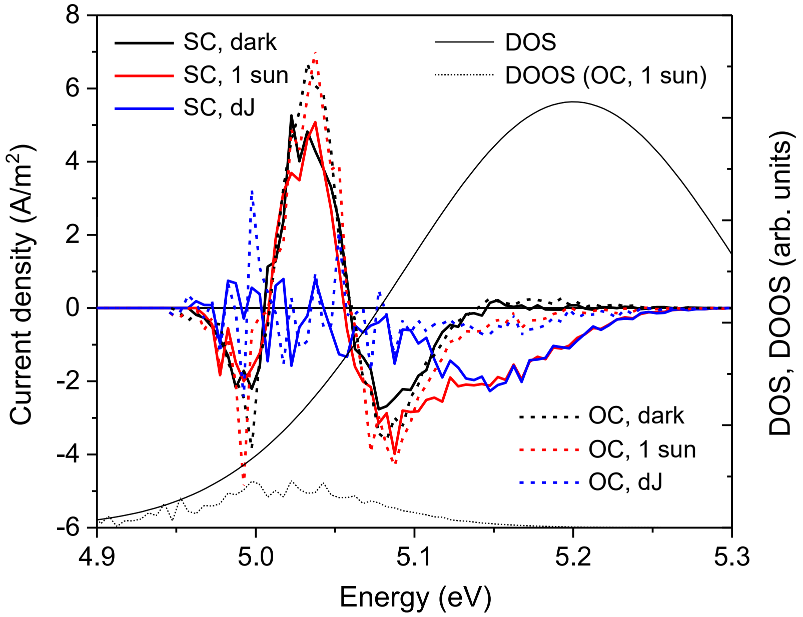

Figure S6: Energy-resolved hole currents for TQ1:$PC_{71}BM$ w.r.t. the HOMO DOS. The solid thick lines show KMC-calculated current densities under short circuit (SC, solid) and open circuit (OC, dashed) conditions, the blue lines are the differences between the currents in the dark (black) and under illumination (red). The thin solid and dashed lines indicate the density of states (DOS) and density of occupied states (DOOS) for reference.

5 Current–Voltage Curves at Large Reverse Bias

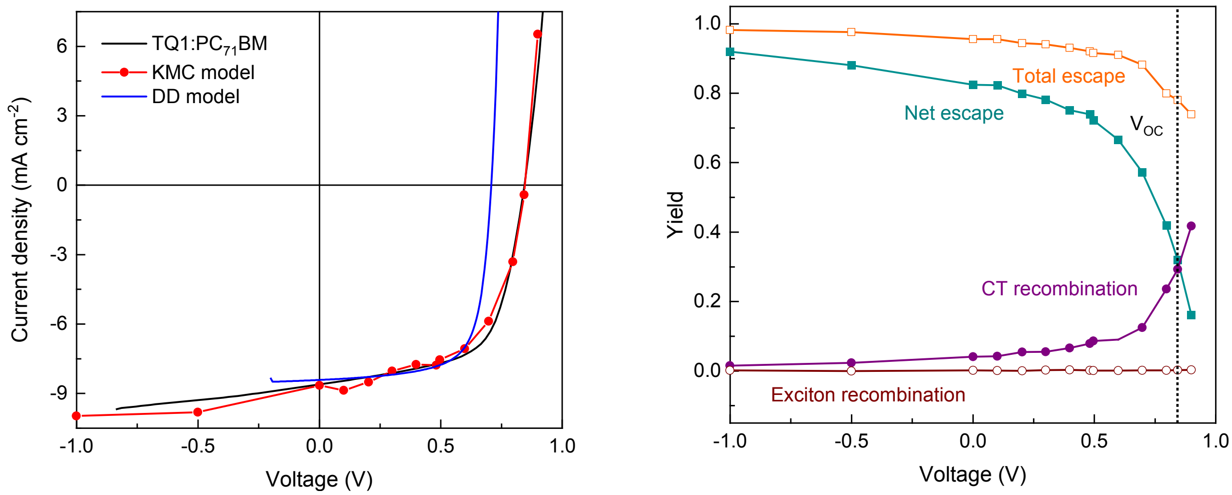

Figure S7: Measured versus modelled J–V curves and loss analysis. Same data as in Fig. 1 of the main text for a wider revers bias range. (a) The black line represents measured J–V characteristics of a 70-nm thick TQ1:PC$_{71}$BM solar cell under illumination. Only the KMC model (red symbols) reproduces the gradual increase in current density towards larger reverse bias, as observed in the experiment. The DD model (blue line) predicts a constant current beyond $V = 0$. The KMC and DD models use a single, consistent set of parameters. (b) Corresponding extraction and loss yields from KMC. Total and net escape yields are defined as $y_{\text{total}} = (J_{n,\text{an}}+J_{n,\text{cat}}+J_{p,\text{an}}+J_{p,\text{cat}})/J_{\text{abs}}$ and $y_{\text{net}} = (-J_{n,\text{an}}+J_{n,\text{cat}}+J_{p,\text{an}}-J_{p,\text{cat}})/J_{\text{abs}}$, where $J_{(n/p),(\text{an}/\text{cat})}$ is the current density of photogenerated electrons/holes extracted via the anode/cathode and J_{abs} is the current density corresponding to light absorption. The curves labelled exciton and CT recombination show the relative current densities associated with exciton and CT recombination, i.e., the fraction of photogenerated charges that undergo these processes.

6 Temperature Dependent Drift–Diffusion Simulations

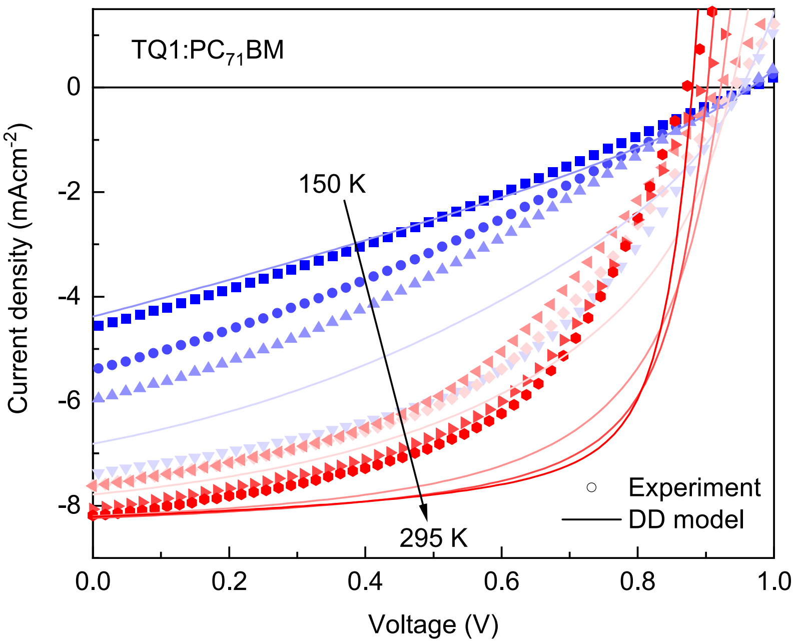

Figure S8: Temperature dependent J–V characteristics for a 75-nm TQ1:PC$_{71}$BM device (symbols, same data as in Fig. 4a of the main text) and DD simulations (lines) with the parameters from Table S2. The simulations have been shifted to match V_{OC}.

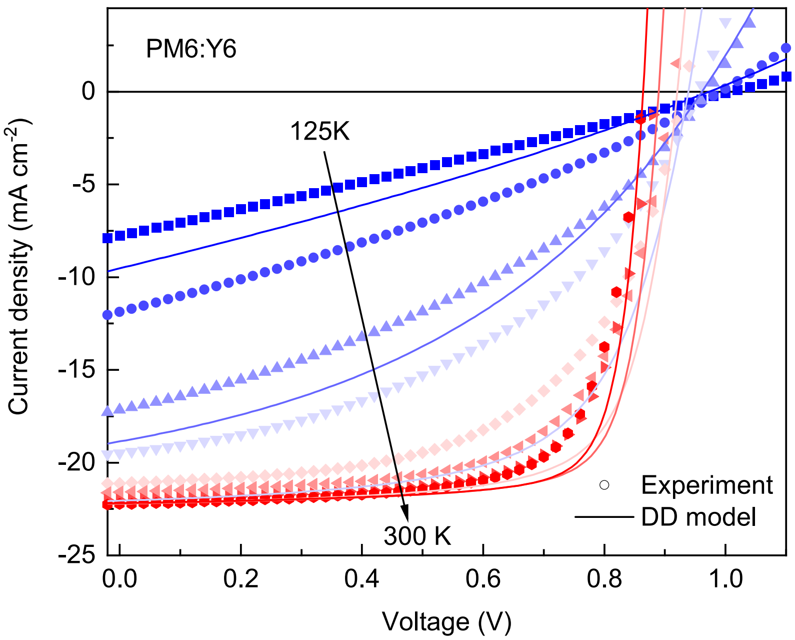

Figure S9: Temperature dependent J–V characteristics for a 115-nm PM6:Y6 device (symbols, same data as in Fig. 4c of the main text) and DD simulations (lines) with the parameters from Table S2 that have been shifted to match V_{OC}. Note the failure of the latter to describe the measured evolution of the fill factor with temperature.

7 Temperature Dependence for a Thick TQ1:$PC_{71}BM$ Device

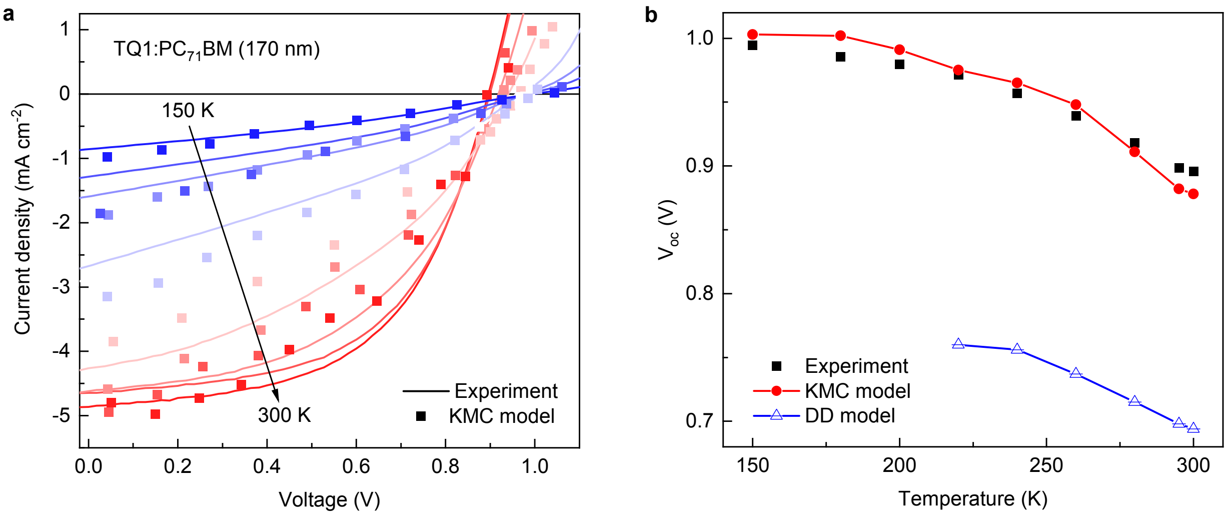

Figure S10: (a) Measured and KMC-modeled temperature dependent J–V characteristics for a 170-nm thick TQ1:$PC_{71}BM$ solar cell. (b) Corresponding V_{OC} at different temperature compared with the DD model. Due to numerical instabilities, the DD model did not converge below ∼230 K. Note in this figure that the (forward) bias dependence of the J–V curves around room temperature is suppressed as compared to the thin device shown in Figs. 1 and 4 of the main text and in Figure S7 above. This is consistent with the interpretation in terms of a diffusion loss due to highly diffusive 'hot' charge carriers: for thicker devices, a smaller fraction of carriers is generated within the diffusion distance from the contact. Consequently, the agreement of DD simulations with experiments at large reverse bias improves for thicker devices around room temperature.

8 Role of Energetic Disorder

Comparing the predictions from the KMC model with those from equilibrium models is complicated by the fact that the bimolecular recombination rate is emergent in KMC but must be explicitly parametrized in equilibrium models as those by Blakesley and Neher[S21] or drift–diffusion; typically a reduced Langevin rate is used. Since the actual reduction value depends critically on essentially all KMC parameters, including disorder, the equilibrium V_{OC} prediction becomes somewhat arbitrary. Therefore, Figure S11 shows two limiting cases: a constant low bimolecular recombination rate ($k_2 = 2 \times 10^{-17}\,\text{m}^3\text{s}^{-1}$ from Table S2, green dashed line) and the Langevin value ($k_L = q/\varepsilon_0\varepsilon_r(\mu_e + \mu_h)$ with mobilities $\mu_{e,h}$ that correspond to the used disorder and hopping parameters, blue dashed line). Since the actual bimolecular recombination rate is significantly reduced relative to the Langevin value due to the morphology and re-splitting of interfacial CT states, the former curve is the more relevant one for the current system. In line with the finding in the main text that the difference between KMC and DD is larger for the more disordered TQ1:PC_{71}BM system than for the less disordered PM6:Y6 system (0.17 V vs. 0.13 V, see Fig. 4 in the main text), the difference with the nonequilibrium KMC model becomes smaller for lower disorder values.

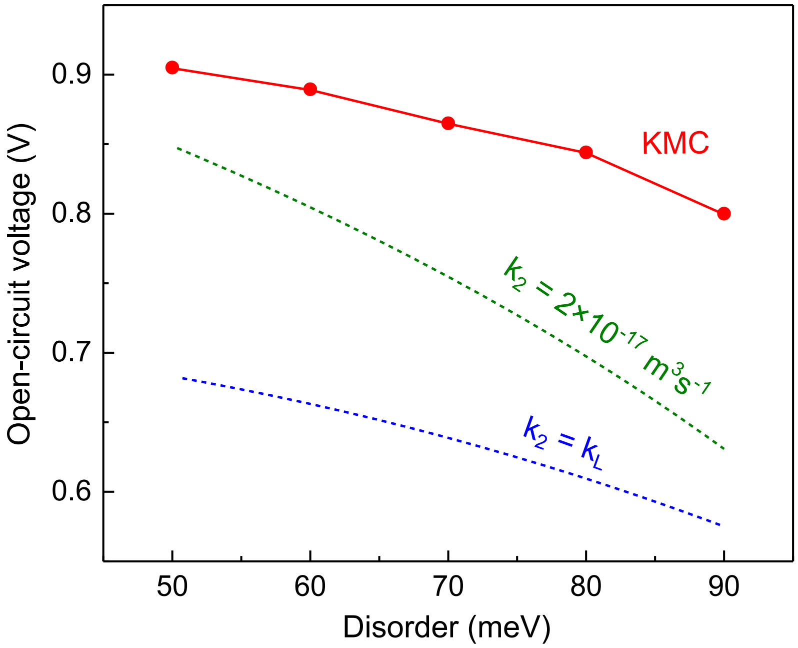

Figure S11: Role of disorder on the difference between equilibrium and nonequilibrium models. Symbols indicate the results of KMC simulations using the parameters for the PM6:Y6 system (Table S1) but with varying energetic disorder σ. The lines are the predictions from the equilibrium model by Blakesley and Neher[S21] for the same parameters, using either the constant low bimolecular recombination rate from Table S2 (green dashed line) or the Langevin value (blue dashed line).

9 Yields for Drift–Diffusion

Figure S12 shows the yields for charge extraction and recombination from the different models. Comparing the KMC (symbols) and DD (solid lines) simulations, one notices that the bias dependence is stronger in the latter case, especially for the recombination. For both KMC and DD, the upswing in recombination (red lines) around V_{OC} is strongly affected by recombination of charges that are injected from the contacts. As argued in the main text, the voltage dependence of the escape curve (black lines) for KMC largely reflects increasing diffusion losses. The limited information that can be extracted from the DD simulation does not allow a similar assignment, but the strong and field dependent recombination suggests that, especially around V_{OC}, the losses are mostly due to recombination. Note also that slightly beyond V_{OC} the escape yield for DD becomes negative, indicating that all photogenerated charges recombine, which is not observed in KMC. As such, the fact that the escape yields for KMC and DD are roughly the same at exactly open-circuit conditions should not be overinterpreted as a sign of equivalence of the two models.

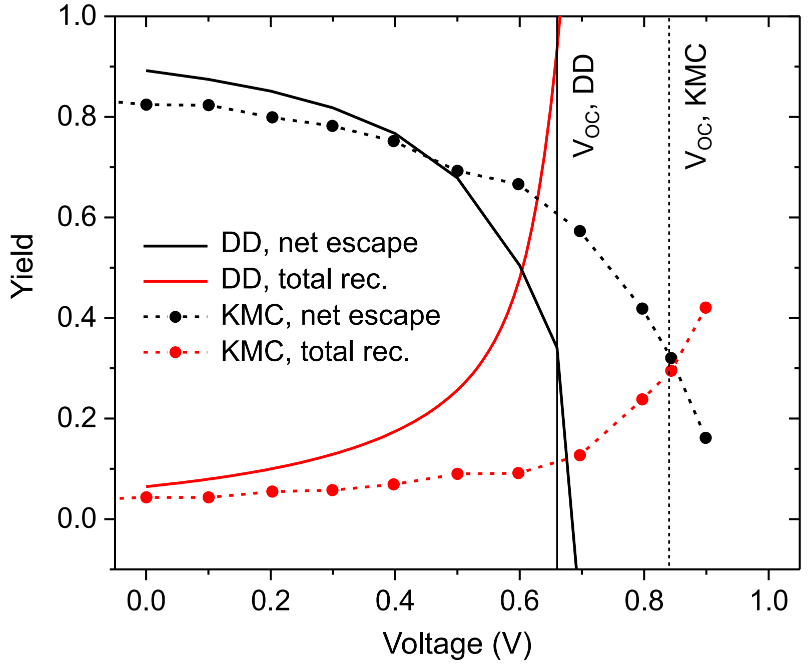

Figure S12: Comparison between recombination and escape yields from KMC and DD models. Since the DD formalism does not allow to label charges as being photogenerated or injected from the contacts but instead works with a single distribution per type of charge carrier (electrons or holes), one cannot produce an equivalent graph to Fig. 1b in the main text. Likewise, there is only a single recombination rate in drift–diffusion. However, one can define a total recombination yield as the recombination current normalized to the maximum photocurrent $J_{\mathrm{gen}} = qGd$ and compare this to the total (CT + exciton recombination = geminate + nongeminate) recombination from KMC. Likewise, one can use the methodology from Kirchartz et al.[S15] to determine a net escape yield from DD.

References

(S1) Wang, E.; Hou, L.; Wang, Z.; Hellström, S.; Zhang, F.; Inganäs, O.; R., A. M. An Easily Synthesized Blue Polymer for High-Performance Polymer Solar Cells. *Adv. Mater.* **2010**, *22*, 5240–5244.

(S2) Yuan, J.; Zhang, Y.; Zhou, L.; Zhang, G.; Yip, H. L.; Lau, T. K.; Lu, X.; Zhu, C.; Peng, H.; Johnson, P. A.; Leclerc, M.; Cao, Y.; Ulanski, J.; Li, Y.; Zou, Y. Single-Junction Organic Solar Cell with over 15% Efficiency Using Fused-Ring Acceptor with Electron-Deficient Core. *Joule* **2019**, *3*, 1140–1151.

(S3) Felekidis, N.; Melianas, A.; Kemerink, M. The Role of Delocalization and Excess Energy in the Quantum Efficiency of Organic Solar Cells and the Validity of Optical Reciprocity Relations. *J. Phys. Chem. Lett.* **2020**, *11*, 3563–3570.

(S4) Wilken, S.; Upreti, T.; Melianas, A.; Dahlström, S.; Persson, G.; Olsson, E.; Österbacka, R.; Kemerink, M. Experimentally Calibrated Kinetic Monte Carlo Model Reproduces Organic Solar Cell Current-Voltage Curve. *Sol. RRL* **2020**, *4*, 2000029.

(S5) van der Holst, J. J. M.; Uijttewaal, M. A.; Ramachandhran, B.; Coehoorn, R.; Bobbert, P. A.; de Wijs, G. A.; de Groot, R. A. Modeling and Analysis of the Three-Dimensional Current Density in Sandwich-Type Single-Carrier Devices of Disordered Organic Semiconductors. *Phys. Rev. B* **2009**, *79*, 085203.

(S6) Felekidis, N.; Melianas, A.; Kemerink, M. Automated Open-Source Software for Charge Transport Analysis in Single-Carrier Organic Semiconductor Diodes. *Org. Electron.* **2018**, *61*, 318–328.

(S7) Zhan, L.; Li, S.; Lau, T.-K.; Cui, Y.; Lu, X.; Shi, M.; Li, C.-Z.; Li, H.; Hou, J.; Chen, H. Over 17% Efficiency Ternary Organic Solar Cells Enabled by Two Non-Fullerene Acceptors Working in an Alloy-like Model. *Energy Environ. Sci.* **2020**, *13*, 635–645.

(S8) Upreti, T.; Wang, Y.; Zhang, H.; Scheunemann, D.; Gao, F.; Kemerink, M. Experimentally Validated Hopping-Transport Model for Energetically Disordered Organic Semiconductors. *Phys. Rev. Applied* **2019**, *12*, 064039.

(S9) Scharfetter, D. L.; Gummel, H. K. Large-Signal Analysis of a Silicon Read Diode Oscillator. *IEEE Trans. Electron Devices* **1969**, *16*, 64–77.

(S10) Roichman, Y.; Tessler, N. Generalized Einstein Relation for Disordered Semiconductors—Implications for Device Performance. *Appl. Phys. Lett.* **2002**, *80*, 1948–1950.

(S11) Paasch, G.; Scheinert, S. Charge Carrier Density of Organics with Gaussian Density of States: Analytical Approximation for the Gauss–Fermi Integral. *J. Appl. Phys.* **2010**, *107*, 104501.

(S12) Pasveer, W. F.; Cottaar, J.; Tanase, C.; Coehoorn, R.; Bobbert, P. A.; Blom, P. W. M.; de Leeuw, D. M.; Michels, M. A. J. Unified Description of Charge-Carrier Mobilities in Disordered Semiconducting Polymers. *Phys. Rev. Lett.* **2005**, *94*, 206601.

(S13) Kniepert, J.; Paulke, A.; Perdigón-Toro, L.; Kurpiers, J.; Zhang, H.; Gao, F.; Yuan, J.; Zou, Y.; Le Corre, V. M.; Koster, L. J. A.; Neher, D. Reliability of Charge Carrier Recombination Data Determined with Charge Extraction Methods. *J. Appl. Phys.* **2019**, *126*, 205501.

(S14) Burkhard, G. F.; Hoke, E. T.; McGehee, M. D. Accounting for Interference, Scattering, and Electrode Absorption to Make Accurate Internal Quantum Efficiency Measurements in Organic and Other Thin Solar Cells. *Adv. Mater.* **2010**, *22*, 3293–3297.

(S15) Kirchartz, T.; Nelson, J.; Rau, U. Reciprocity between Charge Injection and Extraction and Its Influence on the Interpretation of Electroluminescence Spectra in Organic Solar Cells. *Phys. Rev. Appl.* **2016**, *5*, 054003.

(S16) Puttisong, Y.; Xia, Y.; Chen, X.; Gao, F.; Buyanova, I. A.; Inganäs, O.; Chen, W. M. Charge Generation via Relaxed Charge-Transfer States in Organic Photovoltaics by an Energy-Disorder-Driven Entropy Gain. *J. Phys. Chem. C* **2018**, *122*, 12640–12646.

(S17) Melianas, A.; Etzold, F.; Savenije, T. J.; Laquai, F.; Inganäs, O.; Kemerink, M. Photo-Generated Carriers Lose Energy During Extraction from Polymer-Fullerene Solar Cells. *Nat. Commun.* **2015**, *6*, 8778.

(S18) Melianas, A.; Pranculis, V.; Devižis, A.; Gulbinas, V.; Inganäs, O.; Kemerink, M. Dispersion-Dominated Photocurrent in Polymer:Fullerene Solar Cells. *Adv. Funct. Mater.* **2014**, *24*, 4507–4514.

(S19) Felekidis, N.; Melianas, A.; Aguirre, L. E.; Kemerink, M. Comment on "Charge Carrier Extraction in Organic Solar Cells Governed by Steady-State Mobilities". *Adv. Energy Mater.* **2018**, *8*, 1800419.

(S20) Melianas, A.; Felekidis, N.; Puttisong, Y.; Meskers, S. C. J.; Inganäs, O.; Chen, W. M.; Kemerink, M. Nonequilibrium Site Distribution Governs Charge-Transfer Electroluminescence at Disordered Organic Heterointerfaces. *Proc. Natl. Acad. Sci. USA* **2019**, *116*, 23416–23425.

(S21) Blakesley, J. C.; Neher, D. Relationship Between Energetic Disorder and Open-Circuit Voltage in Bulk Heterojunction Organic Solar Cells. *Phys. Rev. B* **2011**, *84*, 075210.
